# Five millennia of mitonuclear discordance in Atlantic bluefin tuna identified using ancient DNA

**DOI:** 10.1038/s41437-025-00745-1

**Published:** 2025-02-07

**Authors:** Emma Falkeid Eriksen, Adam Jon Andrews, Svein Vatsvåg Nielsen, Per Persson, Estrella Malca, Vedat Onar, Veronica Aniceti, Gäel Piquès, Federica Piattoni, Francesco Fontani, Martin Wiech, Keno Ferter, Oliver Kersten, Giada Ferrari, Alessia Cariani, Fausto Tinti, Elisabetta Cilli, Lane M. Atmore, Bastiaan Star

**Affiliations:** 1 Centre for Ecological and Evolutionary Synthesis (CEES), Department of Biosciences (IBV), University of Oslo, Oslo, Norway; 2 Norwegian Institute of Water Research, Oslo, Norway; 3 Department of Biological, Geological and Environmental Sciences, University of Bologna, Ravenna, Italy; 4 Stavanger Maritime Museum, Stavanger, Norway; 5 Museum of Cultural History, University of Oslo, Oslo, Norway; 6 Cooperative Institute for Marine and Atmospheric Studies, University of Miami, Miami, FL USA; 7 NOAA Fisheries, Southeast Fisheries Science Center, Miami, FL USA; 8 Milas Faculty of Veterinary Medicine, Muğla Sıtkı Kocman University, Muğla, Türkiye; 9 Consejo Superior de Investigaciones Científicas, Institució Milà i Fontanals (CSIC-IMF), Barcelona, Spain; 10 ASM, CNRS, Université Paul Valéry-Montpellier 3, Montpellier, France; 11 Department of Cultural Heritage, University of Bologna, Ravenna, Italy; 12 Institute of Marine Research, PO Box 1870, N-5817 Bergen, Norway

**Keywords:** Genome evolution, Evolutionary genetics

## Abstract

Mitonuclear discordance between species is readily documented in marine fishes. Such discordance may either be the result of past natural phenomena or the result of recent introgression from previously seperated species after shifts in their spatial distributions. Using ancient DNA spanning five millennia, we here investigate the long-term presence of Pacific bluefin tuna (*Thunnus orientalis*) and albacore (*Thunnus alalunga*) -like mitochondrial (MT) genomes in Atlantic bluefin tuna (*Thunnus thynnus*), a species with extensive exploitation history and observed shifts in abundance and age structure. Comparing ancient (*n* = 130) and modern (*n* = 78) Atlantic bluefin MT genomes from most of its range, we detect no significant spatial or temporal population structure, which implies ongoing gene flow between populations and large effective population sizes over millennia. Moreover, we identify discordant MT haplotypes in ancient specimens up to 5000 years old and find that the frequency of these haplotypes has remained similar through time. We therefore conclude that MT discordance in the Atlantic bluefin tuna is not driven by recent introgression. Our observations provide oldest example of directly observed MT discordance in the marine environment, highlighting the utility of ancient DNA to obtain insights in the long-term persistence of such phenomena.

## Introduction

Discordance between mitochondrial (MT) and nuclear gene phylogenies is commonly observed in eukaryotes and can result from incomplete lineage sorting (ILS) or introgression from another species (Kimball et al. 2021; Tamashiro et al. 2019; Platt et al. 2018). Although frequency of the phenomenon across biological systems remains debated, the increased use of next-generation sequencing across non-model taxa has revealed mitonuclear discordance to be a more common phenomenon in nature than previously thought (Dagilis et al. 2022). The majority of documented mitonuclear discordance in animals has been explained as the result of introgression from a closely related species (Sloan et al. 2017; Pons et al. 2014; Toews and Brelsford 2012). Typically inherited maternally in vertebrates, the non-recombining introgressed MT genome remains largely intact over time (Seixas et al. 2018; Brown 2008). The presence of introgressed MT haplotypes can cause significant bias when using mitogenomic data to describe a species demographic properties or evolutionary history. Even rare hybridization events can result in the presence of whole MT haplotypes that do not accurately reflect the typical history or demography of the taxon. For example, the presence of introgressed MT haplotypes may dominate genealogies with recent dispersal history and thereby overshadow genetic signals from past dispersal events (Sloan et al. 2017; Ballard and Whitlock 2004). Presence of heterospecific haplotypes will also affect population genomic analyses by inflating measures of genetic diversity and divergence (Oosting et al. 2023; Rodriguez and Krug 2022; Wang et al. 2022; Hawks 2017). Avoiding such inflation is important because these statistics can influence management choices (Willi et al. 2022; Hohenlohe et al. 2021; Kardos et al. 2021) and increased measures of genetic diversity or effective population size may exaggerate the genetic robustness of a truly vulnerable population.

Marine fish hybridize according to their ecologies and life history strategies, thus the rate of hybridization and proportion of introgression will vary according to migration behaviour, spawning site overlap, fecundity, spawning ontology, and offspring survival (Montanari et al. 2016; Gardner 1997; Hubbs 1955). In the economically important redfish (*Sebastes spp*.), high proportions of introgressive hybridization (15% of all samples) have been found between two species (*S. fasciatus* and *S. mentella*) that live sympatrically in hybrid zones and yet maintain their morphology, resembling one of the parent species (Benestan et al. 2021; Roques et al. 2001). Likewise, introgression has been observed in European seabass (*Dicentrarchus labrax*) (Duranton et al. 2020; Vandeputte et al. 2019), capelin (Mallot*us villosus*) (Cayuela et al. 2020; Colbeck et al. 2011), European anchovy (*Engraulis encrasicolus*) (Le Moan et al. 2016), Australasian snapper (Chrysophrys auratus) (Oosting et al. 2023) and Atlantic and Pacific herring (Clu*pea harengus* and *C. pallasii*) (Semenova 2020).

Formation of hybrid zones after recent range shifts induced by contemporary climate change have already been observed in a number of species (Kersten et al. 2023; Ottenburghs 2021; Taylor and Larson 2019; Ryan et al. 2018; Garroway et al. 2010) including marine fish (Muhlfeld et al. 2014; Potts et al. 2014). The formation of such hybrid zones can have both deleterious and advantageous effects. For instance, in trout, warmer freshwater temperatures and lower precipitation is expected to increase introgressive hybridization between native European brown trout (*Salmo trutta*) and released non-native brown trout in Mediterranean rivers, potentially leading to loss of local genetic variants (Vera et al. 2023). Yet in rainbowfish (Melanotaenia spp.), it has been suggested that introgressive hybridization contributes to climate change resiliency by incorporating potentially adaptive genetic variation (Brauer et al. 2023; Turbek and Taylor 2023). Regardless of the evolutionary consequences, knowledge about the *timing* of the introgression is necessary to understand if it is anthropogenic impacts that increase rates of hybridization, thereby positively or negatively altering the adaptive potential of species (Xuereb et al. 2021; Hoffmann and Sgrò 2011).

Atlantic bluefin tuna (*Thunnus thynnus*, Linneaus 1758) is a highly migratory marine predatory fish distributed across the Atlantic Ocean (SCRS 2023; Nøttestad et al. 2020; Block 2019). Atlantic bluefin exhibits strong natal homing behaviour (Brophy et al. 2016; Boustany et al. 2008; Block et al. 2005) and is therefore managed as two separate stocks: the larger Eastern stock spawning predominantly in the Mediterranean, and a smaller Western stock spawning predominantly in the Gulf of Mexico (ICCAT 2023). Recent studies, however, have demonstrated weak genetic divergence in Atlantic bluefin and the existence of a previously unknown spawning ground in the Slope Sea where the stocks seem to interbreed (Diaz-Arce et al. 2024; Aalto et al. 2023; Andrews et al. 2021; Rodríguez‐Ezpeleta et al. 2019), thereby challenging the assumption of two reproductively isolated populations. After severe international overfishing during the last century, the Eastern Atlantic bluefin stock has at present recovered due to strict management measures and favourable oceanographic conditions in the recent decade (ICCAT 2022a, 2022b) followed by improved recruitment with a series of very strong year classes (i.e. *individuals born during the same spawning season)* (ICCAT 2023; Reglero et al. 2018; Garcia et al. 2013). Nonetheless, the heavy exploitation (Andrews et al. 2022; Block 2019; MacKenzie et al. 2009), lead to shifts in its age structure and foraging behaviour (Andrews et al. 2023a; Di Natale 2015; MacKenzie et al. 2014; Worm and Tittensor 2011). These distributional changes, as well as the establishment of potentially new spawning grounds may impact the potential for introgression between different species.

The phylogeny within the *Thunnus* genus has been debated and was only recently resolved (Díaz-Arce et al. 2016; Santini et al. 2013; Viñas and Tudela 2009; Chow et al. 2006; Alvarado Bremer et al. 1997; Chow and Kishino 1995). The Atlantic bluefin was previously thought to be a subspecies of Northern bluefin tuna together with Pacific bluefin (*Thunnus orientalis*, Temminck and Schlegel 1844). The bluefins are now regarded as distinct species forming a monophyletic group (Ciezarek et al., (2019); Díaz-Arce et al. 2016; Chow et al. 2006) (see Fig. S1), with non-overlapping ranges (Tseng et al. 2011), with the albacore tuna (Thunnus alal*unga*, Bonnaterre 1788) consistently appearing as sister-species. Yet in mitochondrial phylogenies, the Pacific bluefin is more closely related to albacore tuna (Gong et al. 2017; Viñas and Tudela 2009; Chow et al. 2006) than the Atlantic bluefin. Albacore tuna is found in both the Pacific, Indian and Atlantic Oceans, including the Mediterranean Sea, typically preferring warmer waters than the Pacific and Atlantic bluefins, but with largely overlapping ranges and spawning areas (Saber et al. 2015; Chow and Ushiama 1995).

Pacific bluefin- and albacore-like MT genomes have been observed in the Atlantic bluefin and Atlantic bluefin- and albacore-like MT genomes have been observed the Pacific bluefin, but no bluefin-like MT genomes have been found in albacore (e.g. Diaz-Arce et al. 2024; Chow and Kishino 1995). The presence of the discordant MT genomes has been explained by introgression (Viñas et al. 2003,2011; Viñas and Tudela 2009; Rooker et al. 2007; Chow et al. 2006; Alvarado Bremer et al. 2005; Carlsson et al. 2004; Chow and Kishino 1995; Chow and Inoue 1993). In the Atlantic bluefin, the rates of Pacific bluefin- and albacore-like MT genomes are similar at around 2–5% (Viñas and Tudela 2009; Rooker et al. 2007). Nonetheless, it is unclear if these rates are stable over longer periods of time. In addition to recent distributional shifts likely caused by high fishing pressures, it is possible that climate warming has contributed to novel opportunities for introgression in recent decades. The distribution of Atlantic bluefin over the last century has fluctuated with temperature (Faillettaz et al. 2019; Ravier and Fromentin 2004), and ocean warming has been implicated in altering migration patterns, spawning ontology, and habitats of the Atlantic bluefin (Diaz-Arce et al. 2024; Fiksen and Reglero 2022; Faillettaz et al. 2019; Muhling et al. 2011). Determining the frequency of discordant MT genomes in the past can therefore shed light on the drivers of such phenomena in modern populations.

**Fig. 1:**
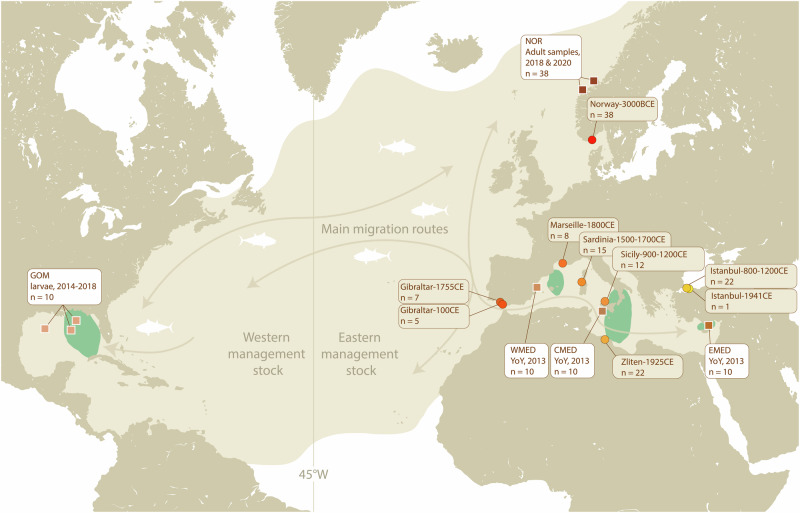
Distribution of the Atlantic bluefin tuna, including spawning areas (green) currently considered by management (adapted from IMR (2021)). The equal-distance line (45°W) separates the Eastern and Western stocks for management purposes. Sample locations of modern (squares, white boxes) and ancient tuna (circles, brown boxes) used in this study are indicated on the map. Arrows indicate the main migration routes of adult Atlantic bluefin (adapted from Fromentin et al. 2014). GOM Gulf of Mexico, NOR Norway, WMED Western Mediterranean, CMED Central Mediterranean, EMED Eastern Mediterranean. YoY young-of-the-year.

Here, we use DNA extracted from ancient Atlantic bluefin specimens to directly investigate the past occurance of MT discordance and to elucidate potential changes in population structure and genetic diversity over time (Kersten et al. 2023). Fish bones have physiological qualities that may increase the likelihood of finding well preserved DNA (Ferrari et al. 2021; Kontopoulos et al. 2019; Szpak 2011) allowing for whole genome sequencing (Star et al. 2017), even from very limited amounts of bone (e.g. <10 mg) (Atmore et al. 2023). Here we use such ancient DNA (aDNA) methods to analyze MT genomes from 130 ancient and 78 modern Atlantic bluefin spanning a period of approximately 5000 years (Fig. 1). By sampling before and after the period of heavy exploitation (1970–2007) and predating anthropogenic climate change, we investigate spatiotemporal patterns of genetic diversity.

## Methods

### Collection, extraction, and sequencing of ancient samples from Norway

38 Neolithic (ca. 3000 BCE) tuna bones from the south of Norway were obtained from three archaeological excavations at Jortveit from 2018 to 2020. Bones were found at varying depths (42–130 cm) in six of nine total trenches and were estimated to be from 3700–2500 BCE based on radiocarbon dating of wood and charcoal from the sediment profiles, as well as directly dated bone harpoons. Three of the bones were also directly radiocarbon dated to the period approximately 3400–2800 BCE (Nielsen 2020a, 2020b, 2020c; Nielsen and Persson 2020).

All laboratory work prior to PCR was performed in a dedicated aDNA laboratory at the University of Oslo, following strict anti-contamination protocols (Llamas et al. 2017; Gilbert et al. 2005). All samples were extracted using a standard extraction protocol adapted from Dabney et al. (2013) after a pre-digestion step (DD from Damgaard et al. 2015) or mild bleach treatment and pre-digestion (BleDD from Boessenkool et al. 2017) as described in Ferrari et al. (2021) (Table S1). Dual-indexed sequencing libraries were built as double stranded, blunt-ended libraries following Meyer and Kircher (2010) and Kircher et al. (2011) with modifications or as single stranded libraries following the Santa Cruz Reaction (SCR) protocol (Kapp et al. 2021) (Table S1). Libraries were sequenced on the Illumina HiSeq 4000 or NovaSeq 6000 (SP Flow Cell) platforms at the Norwegian Sequencing Centre with paired-end 150 bp reads and demultiplexed allowing zero mismatches in the index tag. For additional details, see supplementary section 1.2.

### Ancient specimens from the Mediterranean

92 individuals from archaeological excavations and zoological collections throughout the Mediterranean region dating from 100 to 1941 CE were obtained from Andrews et al. (2024) as BAM files (Table S2). For additional details about the samples and archaeological sites, see supplementary section 1.8. These samples were prepared and extracted in the Ancient DNA Laboratory of the Department of Cultural Heritage (University of Bologna, Ravenna Campus, Italy), following strict criteria for aDNA analysis as per the Norwegian samples, and sequenced as single-stranded libraries (Kapp et al. 2021) at Macrogen facilities (Seoul, South Korea/Amsterdam, Netherlands) on a HiSeq X (100 bp paired-end) Illumina sequencing platform. Reads were processed using the Paleomix pipeline v.1.2.14 (Schubert et al. 2014) with settings described below (see “Bioinformatic processing of ancient and modern sequence data”), yielding an average of 28% endogenous DNA and 11-fold MT coverage (Table S8).

### Collection, extraction, and sequencing of modern samples

Modern tuna tissue samples of migratory, foraging adults from Norway (NOR) (*n* = 38) were collected by the Norwegian Institute of Marine Research (IMR), from commercial catch off the coast of Møre og Romsdal, Western Norway (Table S3) (Supplementary section 1.3). The modern samples from Norway were all extracted in the modern DNA isolation laboratories at the University of Oslo, using the DNeasy Blood and Tissue kit (Qiagen) and following the manufacturer’s protocol.

Modern larvae or young-of-the-year (YoY) specimens (GOM: Gulf of Mexico, WMED: Western Mediterranean Balearic Islands, CMED: Central Mediterranean Sicily, EMED: Eastern Mediterranean Levantine Sea, *n* = 40, Table S4) were collected from each of the major Atlantic bluefin spawning sites (Fig. 1) between 2013 and 2018. Juvenile albacore samples from the Bay of Biscay were caught by commercial vessels trolling in the Bay of Biscay between June and September of 2010 (Table S5). Larvae and tissue samples from each specimen were preserved in 96% ethanol and stored at −20 °C until further processing. Modern spawning site and albacore samples were extracted at the University of Bologna by a modified salt-based extraction protocol, as per Cruz et al. (2017), using SSTNE extraction buffer (Blanquer 1990), and treated with RNase to remove residual RNA.

For the Norwegian samples, libraries were built using the TruSeq DNA Nano200 preparation kit (Illumina). Modern spawning site extracts, along with albacore extracts, underwent single stranded library preparation following the SCR library protocol (Kapp et al. 2021). Sequencing and demultiplexing, allowing for zero mismatches, was performed at the Norwegian Sequencing Centre on a combination of the HiSeq 4000 and NovaSeq 6000 (SP Flow Cell) Illumina sequencing platforms with paired-end 150 bp reads for all samples.

Raw sequence data of Pacific bluefin whole genome (Suda et al. 2019) were downloaded from DDBJ (accession no DRA008331) (Table S6) and used for interspecific population structure analyses.

### Bioinformatic processing of ancient and modern sequence data

Both modern and ancient reads were processed using the Paleomix pipeline v.1.2.14 (Schubert et al. 2014). All reads were aligned to a draft nuclear (NCBI BioProject: PRJNA408269) and MT reference genome (GenBank accession nr NC_014052.1) with BWA-mem v.0.7.17 for mapping. Only the MT BAMfiles were further processed in GATK v.4.1.4.0 following GATK best practices (McKenna et al. 2010). Filtered VCFs were indexed using Tabix v.0.2.6 (Li 2011) and consensus sequences created as individual fasta files in BCFtools v.1.9 (bcftools consensus *-H 1*). Outgroup sequences were downloaded from GenBank (Clark et al. 2016) and curated using SeqKit v. 0.11.0 (restart -i) (Shen et al. 2016) so that all sequences started at position 1 in the D-loop, to correspond with the sample sequences. After renaming the fasta headers to their appropriate sample-IDs using BBMap v.38.50b (Bushnell 2014) and combining the files to a multiple sequence alignment (MSA), the joint fasta files were aligned using MAFFT v.7.453 (Katoh et al. 2002) (--auto). For additional details, see supplementary section 1.4.

### Population genomic analyses

After an investigation and creation of datasets (supplementary section 1.5), genetic population structure was investigated using Principal component analyses (PCA). A map of missing loci and base variants diverging from the reference genome, was created to assess missing genotypes in both ancient and modern samples and better visualize introgressed specimens. All plots were created with R 4.3 in RStudio (Rstudio Team 2021), using various packages for data loading, analyses, and visualization (supplementary section 1.1).

Genetic diversity was investigated using a range of standard population genetic measurements (number of haplotypes (Nh), haplotype diversity (hD) number of segregating sites (S), nucleotide diversity (π) (Nei 1987), Tajima’s D (TD) (Tajima 1989), and Fu and Li’s F statistic (F) (Fu and Li 1993)) using Fitchi (Matschiner 2016), DnaSP v.6 (Rozas et al. 2017) and the R-package pegas (Paradis 2010). To account for differences in sample sizes across sites when calculating π and TD, an additional analysis using 1000 bootstrap replicates and subsampling five individuals per round without replacement, was performed in pegas on datasets where the total sample size was over five.

Phylogenetic relationships were investigated using both ML and Bayesian approaches. ML trees with 100 nonparametric bootstrap replicates were created in IQTREE v. 1.6.12 (Nguyen et al. 2015). ModelFinder Plus (MFP) (Kalyaanamoorthy et al. 2017) was used to search all available models, and best-fit models were selected according to the Bayesian Information Criterion (BIC) (Schwarz 1978). Bayesian trees were created in BEAST 2 v.2.6.4 (R. Bouckaert et al. 2014), using the Yule model prior under a strict clock with mutation rate 3.6 × 10^−8^ substitutions per site per year as per Donaldson and Wilson (1999), running MCMC over 800,000,000 generations and sampling once every 1000 generations (supplementary section 1.6). The final trees in all phylogenetic analyses were visualized and curated in FigTree v.1.4.4 (Rambaut 2018).

Evolutionary relationships were visualized using haplotype networks created in Fitchi (*--haploid -p*) using the ML trees generated in IQTREE (described above) (supplementary section 1.7)

Genetic distance between sample locations was assessed using measures of absolute (d_xy_) and relative (ΦST) divergence, calculated using DnaSP v.6 (“*DNA divergence between populations”, all sites*) and Arlequin v.3.5 (Excoffier and Lischer 2010) respectively. In Arlequin, pairwise ΦST was calculated via a distance matrix computed by Arlequin based on Tamura and Nei (1993) and assuming no rate heterogeneity, as suggested by bModelTest (R. R. Bouckaert and Drummond 2017) (implemented in BEAST 2 v.2.6.4 (R. Bouckaert et al. 2014)). To test the significance of ΦST, p-values were generated in Arlequin using 1000 permutations.

## Results

### DNA yield and library success

A total of 1.7 billion sequencing reads were obtained for the 38 ancient samples from Norway. These specimens had remarkable DNA preservation with 100% library success and yielding, on average, 24% endogenous DNA and 20-fold MT coverage (Table S7). The reads showed postmortem degradation patterns expected for authentic aDNA (Fig. S2). A total of 3.1 billion sequencing reads were obtained for the 84 modern specimens, resulting in 711-fold MT coverage on average for the 78 Atlantic bluefin specimens (Tables S9 and S10) and 221-fold MT coverage on average for the six albacore samples (Table S11). The Pacific bluefin raw sequence data from Suda et al. (2019) yielded 3322-fold MT coverage (Table S12). After stringent filtering, 186 out of 208 specimens (~90%) were kept for further analyses (Tables S7, S8).

### Detecting discordant MT genomes

**Fig. 2:**
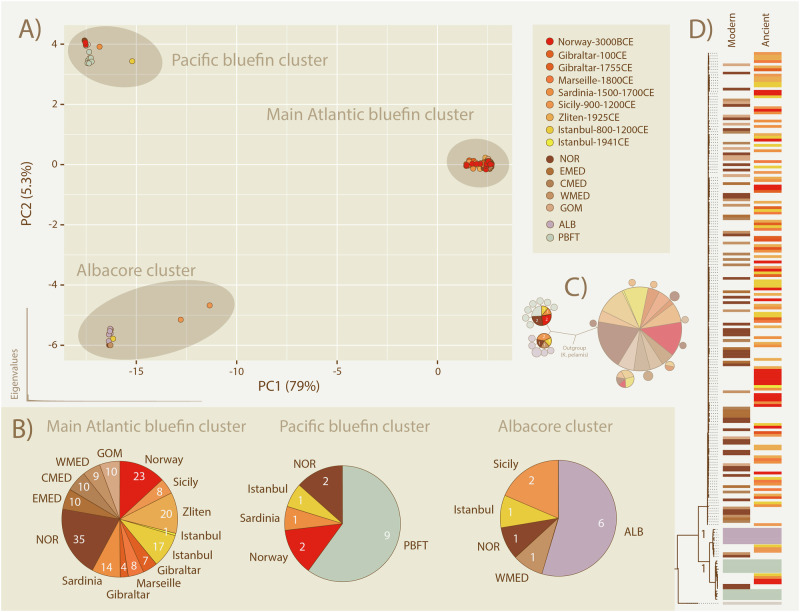
Species clusters and discordant MT haplotypes within Atlantic bluefin specimens, revealed by PCA, haplotype network and phylogenetic analyses. **A** Three species specific clusters detected in ancient and modern Atlantic tuna specimens. The PCA shows three species specific clusters and PCA eigenvalues are shown in the bottom left corner. Modern modern Pacific tuna (blue) and albacore (grey) specimens are included as controls. **B** Relative abundance of haplotypes per location within each PCA-cluster is visualized as pie-charts, with the number of samples from each location indicated on the slices. **C** Haplotype network showing three species specific haplotypes. Haplotypes separated by seven or fewer substitutions were collapsed into single nodes. **D** Interspecific phylogeny of specimens with posterior probability support for the species clades (see also Fig. S6). Colours are representative of the spatiotemporal cohorts listed in the legend of panel (**A**).

Out of 186 samples analyzed, seven ancient and four modern individuals had MT haplotypes that clustered closely with albacore or Pacific bluefin in the PCA, haplotype network and phylogenies. The PCA reveals three distinct clusters (Fig. 2) with PC1 separating an Atlantic bluefin cluster from a Pacific bluefin and albacore cluster and PC2 separating the latter two speciese. Within the Pacific bluefin cluster, we observe two modern (both NOR) and four ancient (two Norway 3000BCE, one Istanbul 800–1200CE and one Sardinia 1500–1700CE) Atlantic bluefin specimens. Within the albacore cluster, we observe two modern (one NOR and one WMED) and three ancient (one Istanbul - 800–1200CE and two Sicily – 900–1200CE) Atlantic bluefin specimens. The PCA-clusters are reiterated in the haplotype network (Fig. 2). ML and Bayesian phylogenetic analyses provided full statistical support (bootstrap = 100, posterior probability = 1) for the three species as monophyletic groups with the same six Pacific-like and five albacore-like haplotypes again clustering with their respective species (Fig. 2, see also Fig. S6).

### Spatiotemporal population structure

We find no significant mitogenomic differentiation between any of the temporal cohorts. We also observe no spatial differences in the level of genetic variation between any of the sampling locations for Atlantic bluefin. Atlantic bluefin individuals from both management stocks and across the Eastern stock range and spawning areas are scattered across the intraspecific PCA (Fig. S8) and haplotype network (Fig. S9). The sampling locations are also distributed along the entire phylogeny within the Atlantic bluefin group (Fig. 2, see also Figs. S6, S10). The intraspecific Atlantic bluefin haplotype network reveals a star-like pattern with more recent MT haplotypes deriving from an ancestral, central haplotype (Fig. S9).

### Genetic divergence and diversity influenced by introgression

**Fig. 3:**
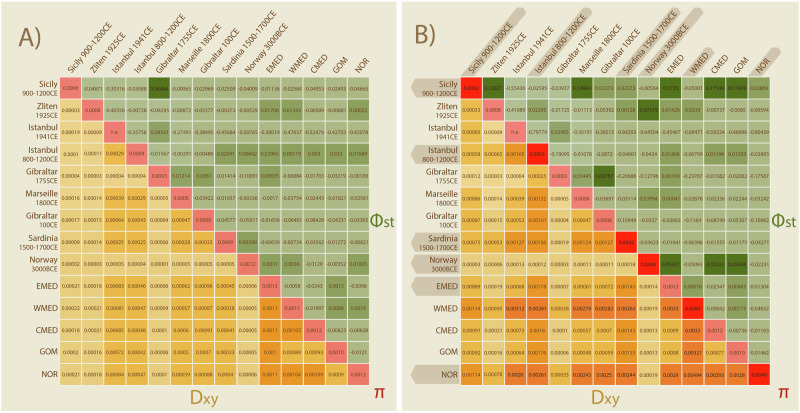
No significant spatiotemporal population structure in Atlantic bluefin tuna based on mitogenomic data of 186 specimens. Pairwise population divergence is presented as a heatmap showing absolute (d_xy_) and relative (ΦST) divergence between populations when (**A**) excluding and (**B**) including the discordant MT genomes. Divergence is increased when including discordant MT genomes. Locations containing discordant MT genomes are highlighted with darker shading in panel (**B**). The nucleotide diversity within each population is shown on the diagonal. P-values for ΦST can be found in supplementary (Fig. S7).

Measures of pairwise genetic distance between Atlantic bluefin sampling locations show low levels of absolute (d_xy_) and relative (Φst) divergence, either excluding (Fig. 3a) or including discordant MT haplotypes (Fig. 3b). Genetic differentiation increases when divergent MT haplotypes are included, which are not present at each location or temporal cohort. In all cases, levels of Φst remained low and non-significant (Fig. S7) across all populations. Including individuals with discordant MT haplotypes increased values of nucleotide diversity π and S (Table S15). The number of haplotypes (hD) is not impacted; most sample locations only contained unique specimens (*N* = Nh) therefore leading to a hD of 1, meaning 100% probability of obtaining unique samples during random sampling. Tajima’s D (TD) was also not affected by the inclusion of introgressed individuals and was significantly negative for most locations and temporal cohorts and when analyzing all specimens jointly (Table S15).

### Frequency of discordant MT haplotypes over time

**Fig. 4:**
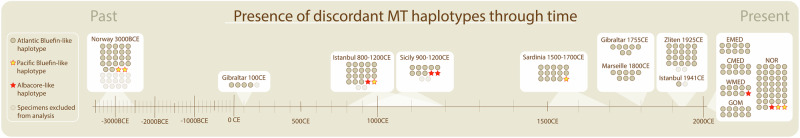
Pacific bluefin-like MT haplotypes (yellow stars) or albacore-like MT haplotypes (red stars) are observed along an entire 5000-year-old chronology of ancient Atlantic bluefin tuna specimens. Individual tuna specimens (*n* = 208) (circle or star) are grouped according to their age, determined by archaeological context. Specimens were either modern (*n* = 78), or ancient (*n* = 130) and dated by archaeological context. Uncertainty in the age range of ancient specimens is depicted beneath their respective sample sets (light shading).

We observe discordant MT genomes in Atlantic bluefin throughout a 5000-year chronology (Fig. 4). The earliest observation is the presence of two Pacific bluefin-like MT genomes in the Neolithic (ca. 3000 BCE) in Norway. Pacific bluefin-like MT genomes are further found in early medieval Istanbul (800–1200 CE), late-medieval Sardinia (1500–1700 CE) and modern Norway. Albacore-like MT haplotypes are found in early medieval Istanbul (800–1200 CE) and Sicily (900–1200 CE), modern Western Mediterranean and modern Norway.

## Discussion

We here present a 5000-year chronology of MT discordance in the Atlantic bluefin tuna. The observation of divergent MT haplotypes in the Neolithic (ca. 3000 BCE), turn of the millennium (800–1200 CE), and in present day populations indicates that it is long-term natural phenomena rather than recent spatial shifts that explains their presence. Moreover, our results show that the frequency of MT discordance in the Atlantic bluefin has remained stable over millennia despite shifts in abundance and distribution of Atlantic bluefin populations.

### Evidence of mitonuclear discordance through time

We obtain similar proportions of for Pacific bluefin- and albacore-like MT haplotypes at 2.6% (2/78 individuals) each as reported by previous studies in our modern samples. Including our ancient samples in the calculation, the proportion of MT discordance remains astonishingly stable with 3.2% Pacific bluefin-like haplotypes (6/186) and 2.7% albacore-like haplotypes (5/186) across all samples. In total, six Pacific-like and five albacore-like haplotypes consistently cluster with their respective species through all interspecific analyses. We do not observe albacore-like haplotypes from the Neolithic period, but because of the low frequency of introgression compared to the sample size we speculate this is likely due to sampling stochasticity rather than their lack of presence at the time. Still, it cannot be excluded that Neolithic climate conditions drove albacore populations away from areas inhabited by Atlantic bluefin.

The observation of discordant MT haplotypes relies on the assumption that the sampled archaeological bones used in this study all stem from Atlantic bluefin individuals. The nuclear DNA of the ancient Mediterranean samples used in this study, has been analyzed as part of Andrews et al. (2024) where all samples, including the individuals with diverging MT haplotypes cluster together with the modern Mediterranean and Norwegian Atlantic bluefin individuals. Some of the ancient Norwegian samples from Jortveit were also included in these analyses, however the two individuals with Pacific-like MT genomes were excluded from the analyses due to bad preservation of the nuclear genome. We therefore cannot be certain that these two individuals are in fact Atlantic bluefin vertebrae, as the Pacific and Atlantic bluefin vertebrae are morphologically diffucult to distinguish. However, no migration of Pacific bluefin into the Atlantic Ocean has ever been observed and there is no known range overlap. The optimal temperature range of Pacific bluefin is around 15–20 °C (Kitagawa et al. 2006), while the ocean surface temperature in Neolithic Norway peaked at around 8 °C in mid-august (Risebrobakken et al., (2011)). We therefore find it highly unlikely that these two individuals stem from migratory Pacific bluefin.

Should the mitonuclear discordance be driven by past introgressive hybridization from both albacore and Pacific bluefin, the location and timing of such events remain to be investigated. While the albacore has overlapping ranges and spawning areas with both bluefin species, the Pacific and Atlantic bluefins are geographically separated with no documented migration. The potential migration of Pacific bluefin into the Atlantic Ocean has been hypothesized to occur via the Indian Ocean and following the Agulhas current around the tip of Africa (Alvarado Bremer et al. 2005). Whether this represents a contemporary migration route or a historical process where past, stronger currents might have facilitated admixture is unclear (Alvarado Bremer et al. 2005). The similar proportion of mitonuclear discordance from both albacore and Pacific bluefin and the lack of divergent MT haplotypes in the albacore makes the range-overlapping albacore an unlikely carrier of MT haplotypes between the bluefins in the case of introgression. Given that Pacific bluefin also contains Atlantic bluefin-like MT haplotypes, incomplete lineage sorting (ILS) can also explain their presence. The *Thunnus* genus is thought to have diverged rapidly within the last 6–10 million years, with a more recent speciation of the Pacific and Atlantic bluefins only around 400,000 years ago (Ciezarek et al., (2019); Díaz-Arce et al. 2016; Santini et al. 2013). While introgressive hybridization is the likely origin of albacore-like haplotypes in both bluefin species (Ciezarek et al., (2019)), observed gene-tree versus species-tree discordance did not deviate from expectations under ILS in the same study (Ciezarek et al., (2019)). These results indicate that the observed patterns of Pacific-like MT haplotypes in the Atlantic bluefin population, and vice versa, may be a result of ILS rather than introgressive hybridization. Larger genomic databases are required to furhter delineate between these two hypotheses.

While our historical investigation focuses on the eastern Atlantic with samples from the Mediterranean and Norway, discordant MT genomes from albacore have also been found in the Gulf of Mexico (1% frequency) and the Slope Sea (6% frequency). Because such MT genomes in Atlantic bluefin were first observed in the eastern Atlantic, their presence in the western Atlantic has been hypothesized to be introduced via gene flow from the Mediterranean (Diaz-Arce et al. 2024). An increase in gene flow from the Mediterranean into the Gulf of Mexico and Slope Sea will likely erode genetic differences between the two management stocks (Diaz-Arce et al. 2024). Direct observations of hybridization events within the Western Atlantic bluefin stock have not been made, although albacore is known to spawn across tropical waters including the South-West Sargasso Sea as well as the Mediterranean (NOAA 2023; ICCAT 2016, 2020). Future studies could monitor the frequencies of MT discordance in the Gulf of Mexico to disentangle the recently suggested changes in demographic patterns (Diaz-Arce et al. 2024) and possibly locate the origins of contemporary introgression events.

### Discordant MT haplotypes impact estimates of mitogenomic differentiation

The presence of discordant haplotypes increases measures of genetic diversity (S and π), which is driven by the high number of diverging bases in the introgressed MT genomes (Fig. S5). The inclusion of these diverging haplotypes also influences the measures of genetic divergence (d_xy_ and ΦST) between locations and temporal cohorts, consistently increasing the absolute genetic diversity (d_xy_) and altering the pattern of relative genetic divergence (ΦST). Tajima’s D (TD) was not consistently affected by the inclusion of introgressed individuals, although in some cases TD changed value and lost or attained significance when divergent haplotypes were included (Table S15). Considering these results, one needs to be aware of highly diverging haplotypes when extrapolating population genetic statistics from subsamples of natural populations containing highly diverging haplotypes. The low frequency of divergent haplotypes in Atlantic bluefin causes stochasticity at low sample sizes, and we find that their inclusion inflates population genomic statistics that are commonly used for management and population viability assessments (e.g. Dapporto et al. 2022; Hohenlohe et al. 2021; Zhang et al. 2020).

### Spatiotemporal population structure

We find no significant divergence and no pattern of mitogenomic differentiation between any of the spatial or temporal cohorts. Ancient and modern samples largely intermixed in all analyses, suggesting mitogenomic stability and temporal continuity through time. Similar observations in other species, such as Atlantic cod (*Gadus morhua*) (Martínez-García et al. 2021) and New Zealand snapper (Chrysophrys *auratus*) (Oosting et al. 2023) emphasize the low power of the MT genome to observe spatiotemporal differentiation in wide ranging fish species. The regular presence of identical haplotypes across sampling locations and temporal cohorts emphasizes the lack of mitogenomic variation and informative markers for population structure in this species. Although we cautiously removed identical samples from the same archaeological excavations, the presence of identical samples across cohorts that were processed in different laboratories shows that identical MT haploptypes can be observed regularly.

Population genetic statistics confirmed low mitogenomic variation with no significant divergence between any of the spatiotemporal cohorts and no temporal loss of genetic diversity despite heavy exploitation (Fig. 3A, Table S15). Across datasets, TD was negative and often significant, suggesting an excess of rare variants in the datasets. This is indicative of either positive selection or recent population expansion (Fijarczyk and Babik 2015; Delph and Kelly 2014). Population expansion is further corroborated in the intraspecific haplotype network, where newer haplotypes are derived from a shared central haplotype forming a star-like pattern (Fig. S9). These results highlight robust preservation of the MT genome despite centuries of human exploitation.

## Conclusion

Atlantic bluefin tuna has experienced significant changes in distribution linked to sea surface temperature oscillations during the past centuries (Faillettaz et al. 2019; Muhling et al. 2011; Ravier and Fromentin 2004), alongside intense exploitation (Andrews et al. 2022; Block 2019), biomass depletion, range contraction, trophic niche loss (Andrews et al. 2021; Di Natale 2015; Tangen 2009), followed by recovery, increased biomass, and range expansion of the Eastern Atlantic bluefin stock during the last decade (ICCAT 2023; Nøttestad et al. 2020). Despite such extensive spatial shifts in distribution over time, we show that the presence of MT discordance is a long-term natural phenomenon in Atlantic bluefin. The stable frequency over time suggests that this phenomenon is robust against recent spatial shifts due to anthropogenic impacts. By providing a baseline observation, our study highlight the utility of aDNA to obtain temporal insights in the long-term persistence of such phenomenon.

## Supplementary information

Supplementary of: Five millennia of mitonuclear discordance in Atlantic bluefin tuna identified using ancient DNA

Supplementary: ENA data

## Data Availability

All mitochondrial BAM files used in this study have been uploaded in the European Nucleotide Archive (ENA) and can be accessed at https://www.ebi.ac.uk/ena/browser/view/PRJEB74135. See supplementary csv containing filenames, project accession number and ENA identifier for all samples.
