# Supplementary material for: Five millennia of mitonuclear discordance in Atlantic bluefin tuna identified using ancient DNA
Source: Heredity (Edinb). 2025 Feb 7;134(3-4):175–85. doi: 10.1038/s41437-025-00745-1 (PMC11977281; doi:10.1038/s41437-025-00745-1)
Supplement: Supplementary file 1 — Supplementary of: Five millennia of mitonuclear discordance in Atlantic bluefin tuna identified using ancient DNA [file 41437_2025_745_MOESM1_ESM.docx]

Supplementary of:

Five millennia of mitonuclear discordance in Atlantic bluefin tuna identified using ancient DNA

Emma Falkeid Eriksen^1*^, Adam Jon Andrews^1,2,3^, Svein Vatsvåg Nielsen^4^, Per Persson^5^, Estrella Malca^6,7^, Vedat Onar^8^, Veronica Aniceti^9^, Gäel Piquès^10^, Federica Piattoni^3^, Francesco Fontani^11^, Martin Wiech^12^, Keno Ferter^12^, Oliver Kersten^1^, Giada Ferrari^1^, Alessia Cariani^3^, Fausto Tinti^3^, Elisabetta Cilli^11^, Lane M. Atmore^1^, Bastiaan Star^1*^

^1^ Centre for Ecological and Evolutionary Synthesis (CEES), Department of Biosciences (IBV), University of Oslo, Norway

^2^ Norwegian Institute of Water Research, Oslo, Norway

^3^ Department of Biological, Geological and Environmental Sciences, University of Bologna, Ravenna, Italy

^4^ Stavanger Maritime Museum, Stavanger, Norway

^5^ Museum of Cultural History, University of Oslo, Norway

^6^ Cooperative Institute for Marine and Atmospheric Studies, University of Miami, Miami, Florida, United States of America

^7^ NOAA Fisheries, Southeast Fisheries Science Center, Miami, Florida, United States of America

^8^ Osteoarchaeology Practice and Research Centre, Faculty of Veterinary Medicine, Istanbul University-Cerrahpaşa, Istanbul, Türkiye

^9^ Museum of Natural History, University of Bergen, Bergen, Norway

^10^ ASM, CNRS, Université Paul Valéry-Montpellier 3, Montpellier, France

^11^ Department of Cultural Heritage, University of Bologna, Ravenna, Italy

^12^ Institute of Marine Research, PO Box 1870, N-5817 Bergen, Norway

*Corresponding authors: [emeriks@uio.no](mailto:emeriks@uio.no), [bastiaan.star@ibv.uio.no](mailto:bastiaan.star@ibv.uio.no)

Supplementary Section 1

1.1: R-packages used in population genomic analyses

- vcfR (data loading) (Knaus & Grünwald, 2017)
- adegenet (ordination analysis) (Jombart, 2008)
- ape (phylogenetic analyses) (Paradis et al., 2004)
- pegas (population genomic statistics) (Paradis, 2010)
- ggplot in tidyverse (visualization) (Wickham et al., 2019)
- gridExtra (visualization) (Auguie and Antonov 2017)
- lemon (visualization) (Edwards 2017)

1.2: Laboratory processing of ancient samples from Norway

Upon introduction to the aDNA lab, bones were brush-cleaned and UV-ed ten minutes on each side to reduce surface DNA contamination. The bones were then cut using an electric dentistry tool with an attached cutting disc in a sterile extraction hood, preserving morphometric landmarks. Cut fragments were crushed using a custom designed stainless-steel mortar as described in Gondek, Boessenkool, and Star (2018). For DNA extraction, powdered bone (2 × 200 mg per sample) was subjected to the DD or BleDD treatment and digested in 1 ml 0.5 M EDTA, 0.5 mg/ml proteinase K and 0.5% N-Laurylsarcosine for 18-24 h at 37 C. Combined digests were extracted with 9 × volumes of PB buffer (QIAGEN) and DNA was purified with MinElute columns on a QIAvac 24 Plus vacuum manifold system (QIAGEN). Meyer and Kircher libraries were built from 20 μL of ligated DNA extract or extraction blanks and performed in half volumes reactions. The single stranded SCR libraries were built from 3-20 μL of ligated DNA extract (depending on the DNA concentration) or 20 μL extraction blanks using dilution tier 4. Indexing PCRs were performed with Taq Pfu Turbo Cx HotStart DNA polymerase (Agilent) with the following cycling conditions: 2 min activation at 95 C, 30 s denaturation at 95 C, 30 s annealing at 60 C, 1 min elongation at 72 C, and 10 min final extension at 72 C. Sample extracts were subject to 12 PCR cycles, while extraction blanks were subject to 30 PCR cycles to increase the chance of detecting contamination. Amplified libraries were cleaned using Agencourt AMPure XP PCR purification beads (Bronner et al., 2013) and examined on a Fragment Analyzer^TM^ (Advanced Analytical) using the High Sensitivity NGS Fragment Analysis Kit to determine suitability for sequencing.

1.3: Collection of modern samples from Norway

Two batches of modern Norwegian samples were obtained, the first from September 2018 and the second from September 2020 (Table S3). These batches were taken from two single purse seine catches and each of them are therefore assumed to belong to the same shoal. The 2018 batch of samples was freeze dried muscle tissue powdered at IMR facilities. The 2020 batch was collected as skin samples cut out between the spines of the dorsal fin and submerged immediately in RNAlater, shipped, and placed in a -20°C freezer within a week.

1.4: Bioinformatic processing of ancient and modern sequence data

Both modern and ancient reads were processed using the Paleomix pipeline v.1.2.14 (Schubert et al., 2014). Adapters were removed and forward and reverse reads were collapsed and trimmed with AdapterRemoval v.2.3.1 (Schubert et al., 2016), discarding collapsed reads shorter than 25 bp. All reads were filtered to a minimum Phred score quality of 25, so that only reads with higher mapping quality to the reference genome were considered endogenous and used for subsequent analyses. PCR duplicates were removed in Picard Tools v.2.18.27 and indel realignment (*GATKs* *IndelRealigner*) was performed to produce final BAM files. DNA post-mortem damage patterns were assessed in mapDamage v.2.0.9 (Ginolhac et al., 2011; Jónsson et al., 2013) after downsampling to 100,000 randomly selected reads. MT BAMfiles were further processed in GATK v.4.1.4.0. Individual genotypes were called (GATK v.4.1.4.0 *HaplotypeCaller -ploidy 1*) and then combined into a joint gvcf (GATK v.4.1.4.0 *CombineGVCFs*) before genotyping (GATK v.4.1.4.0 *GenotypeGVCFs*). Genotypes were hard-filtered in BCFtools v.1.9 (Li et al., 2009a; Li et al., 2009b) (*-i 'FS<60.0 && SOR<4 && MQ>30.0 && QD > 2.0' --SnpGap 10*) and VCFtools v.0.1.16 (Danecek et al., 2011) (*--minGQ 15 --minDP 2 --remove-indels*).

### 1.5: Initial investigations and creation of datasets

Preliminary analyses were performed on a jointly called and filtered VCF and multiple-fasta, which included all samples. Missingness and depth was assessed for all samples using VCFtools v.0.1.16 (Danecek et al., 2011) and Principal Component analyses (PCA) (Adegenet (Jombart, 2008) in R v.4.1.2) and a Maximum Likelihood (ML) tree (IQTREE v. 1.6.12 (Nguyen et al., 2015), -m MFP -bb 1000 -BIC) was used to investigate clustering patterns and assess the presence of discordant MT genomes. To look for identical haplotypes, we assessed the number of single nucleotide polymorphisms (SNPs) that differed between specimens using an in-house python3 script (https://github.com/laneatmore/nucleotide_differences), which uses MSAs as input to count the number of true SNP differences between all individuals (excluding those that were missing data) and generates distance matrices based on these differences.

Given that ancient bones may represent the same individual if they were obtained from the same archaeological context, we assessed the number of pairwise SNP differences between specimens at different filtering settings. To be conservative, ancient samples were considered identical if, in a pairwise comparison, they had no SNP differences at minDP2 or if they had only one SNP difference at minDP3 and came from the same archaeological layer. Using these criteria, we detected several genetically identical specimens of which the specimen with the highest endogenous DNA content was kept. Genetically identical modern samples were kept since distinct individuals were sampled. All samples with missingness above 50% (VCFtools v.0.1.16 --missing-indv, F_MISS) were also discarded. In total, 22 out of 208 samples (~10%) were discarded from further analyses (Table S7 and S8).

Datasets were created for each sampling location depending on the presence of discordant MT haplotypes (Table S13). First, a dataset excluding the discordant haplotypes was created to allow for comparison of the effect of diverging haplotypes on summary statistics. A haplotype was defined to be discordant if it clustered with albacore or Pacific bluefin in the PCA (Figure S3). This clustering was additionally supported by the ML and BEAST trees, which revealed the same individuals falling into highly supported monophyletic clades with the respective species (Figure S4). As the genotyping and filtering process in the GATK pipeline is affected by the haplotype variants present in the analyses, only samples within each dataset should be called and filtered together to accurately present the variation (GATK, 2016). These separate datasets were therefore separately genotyped, filtered, and aligned (with settings described in the section above) to create respective multiple-fasta files for subsequent analyses. An overview of these individual datasets can be found in Table S14.

1.6: Bayesian trees were created in BEAST 2 v.2.6.4. bModelTest (R. R. Bouckaert & Drummond, 2017) was used to assess available site models, and the resulting logfile was inspected in Tracer v1.7.2 (Rambaut et al., 2018). Trees were downsampled in LogCombiner (implemented in BEAST 2 v.2.6.4), resampling every 10,000 trees. TreeAnnotator (implemented in BEAST 2 v.2.6.4) was used to remove the first 10% of the trees (burnin) and create a target maximum clade credibility tree. Nodes with less than 50% posterior support were excluded from the summary analysis in TreeAnnotator so that only nodes present in the majority of the trees were annotated.

1.7: Haplotype networks were created in Fitchi using the ML trees generated in IQTREE as input with bootstrap values removed using the R-package ape (Paradis et al., 2004). For the dataset including discordant haplotypes and outgroup species, a minimum edge length of seven substitutions was defined (*-e 7*) so that haplotypes separated by seven or fewer substitutions were collapsed into one node. For the dataset only containing Atlantic bluefin-like haplotypes, each node was defined as a unique haplotype (*-e 1*).

1.8: Archaeological details for the ancient specimens from the Mediterranean

Gibraltar 100 CE

Five vertebrae were analyzed from the archaeological site ‘Olivillo’, an Imperial Roman fish processing/salting facility within the city of Cádiz (Bernal-Casasola *et al.*, 2020). Specimens were selected from various stratigraphic units, dated by context to the 1st century CE. Specimens were of adult fish ca. 1.5-2m fork length (FL) (Andrews et al. 2023).

Istanbul 800-1200 CE

22 vertebrae were analyzed from a rescue excavation at a Byzantine era site in the Yenikapi neighbourhood of Istanbul, Turkey. The Port of Theodosius operated at this site from 4^th^-11^th^ century CE before being filled in at the 15^th^ century CE (Onar *et al.*, 2008). The 800-1200 CE origin of the samples is proposed from radiocarbon dating by AMS (Accelerator Mass Spectrometry) in a separate study (Andrews et al. 2023). Specimens were adult fish ca. 1.5-2.5m FL.

Sicily 900-1200 CE

12 vertebrae were analyzed from urban layers in three different excavations in settlements in Sicily; Sant’Antonino and Corso dei Mille in Palermo and Mazara del Vallo (southwest Sicily, Italy). The layers were dated by context as detailed in (Aniceti, 2019). Samples were estimated to represent individuals believed to have been caught locally. FL estimates were not made for these individuals as the vertebrae selected were fragmented and could not be assigned to rank or accurately measured. Broadly, specimens represented medium (>1.5m) to large sized adult BFT.

Sardinia 1500-1700 CE

15 vertebrae were analyzed from the archaeological site of ‘Pedras de Fogu’ (Sassari, Sardinia, Italy). Dating was done by context given that a tuna trap (tonnara) operated at this location from the 16th to the end of the 18th century where BFT vertebrae have been recovered in a midden at the back of the beach after they were revealed by coastal erosion (Delussu and Wilkens, 2001). Specimens represented fish ca. 1.5-2m FL (Andrews et al. 2023).

Gibraltar 1755 CE

Seven vertebrae were analyzed from the archaeological site of ‘La Chanca’ (Conil de la Frontera, Spain). La Chanca is a small salting factory belonging to the Duchy of Medina Sidonia that was built in the 16^th^ century and was in operation until the 19th century. On November 1, 1755, an earthquake occurred with an epicentre in the Gulf of Cádiz, which generated a tsunami  in the southeast of Andalusia (Huelva and Cádiz) and along the whole Iberian coast, leaving La Chanca completely destroyed, mid-operation. Excavations (Gutiérrez-Mas *et al.*, 2016) confirmed the strata destroyed by the tsunami, where BFT vertebrae, scales and spines were recovered with an anatomical connection, although separated where presumably BFT were cut into spinal sections in preparation for the salting process. The dating of 1755 is corroborated by a letter from Miguel of Aragón and Serrano, who held the position of Corregidor of Conil in 1755 when he wrote to the Duke of Medina Sidonia detailing that BFT were in the salting piles at the time the catastrophe occurred, that the wave dragged them and that they remained buried under the ruins of the building, with no possibility of recovery (document 2326 of the General Archive of the Medina Sidonia Foundation, Spain). Fork lengths were estimated to range from 1.5-2.2m (Andrews et al. 2023).

Marseille 1800 CE

A total of 8 vertebrae were obtained from the archaeological site of Leca Harbour, Marseille, France which was dated to between the late 18th and early 19th century (Piquès 2020). An approximate date of 1800 CE is shown for these sample groups in analyses. FL estimates were not made for these individuals as the vertebra selected was fragmented and could not be assigned to rank or accurately measured. Specimens represented large ~2m sized adults (Piquès 2020)

Zliten 1925 CE and Istanbul 1941 CE

We analysed vertebrae specimens collected from two locations in the early 20th century by the ecologist Massimo Sella (see Andrews et al. 2023). All specimens consist of vertebrae that were air-dried by the collector after capture and processing at tuna traps (Tonnare). A total of 24 samples were obtained from BFT vertebrae captured in the tonnara at Zilten, Libya (Ionian Sea) in 1925, estimated to represent BFT between 1.5-2m FL. Lastly, two large (ca. 2.75m FL) specimens were sampled that originated from tuna traps in the Bosporus, Istanbul, Turkey in 1941 (Andrews et al. 2023).

Refernce list for supplementary section 1:

Aniceti, V. 2019. Animals and their roles in the medieval society of Sicily: from Byzantines to Arabs and from Arabs to Norman/Swabians. Doctoral Thesis. University of Sheffield.

Andrews, A. J., Pampoulie, C., Di Natale, A., Addis, P., Bernal-Casasola, D., Aniceti, V., Carenti, G., Gómez-Fernández, V., Chosson, V., Ughi, A., Von Tersch, M., Fontanals-Coll, M., Cilli, E., Onar, V., Tinti, F., & Alexander, M. (2023). Exploitation shifted trophic ecology and habitat preferences of Mediterranean and Black Sea bluefin tuna over centuries. *Fish and Fisheries*. https://doi.org/10.1111/faf.12785

Auguie, B., & Antonov, A. (2017). gridExtra: miscellaneous functions for “grid” graphics. R package version, 2(1).

Bernal-Casasola, D., Vargas Girón, J. M., and Lara Medina, Y. (2020). Atunes en salazón y en conserva en las chancas gaditanas: perspectivas desde El Olivillo in 7 metros de la Historia de Cádiz. Darío Bernal, José Manuel Vargas y Macarena Lara (eds.). Arqueología en el Olivillo y en el Colegio Mayor Universitario, 517–534.

Bronner, I. F., Quail, M. A., Turner, D. J., & Swerdlow, H. (2013). Improved Protocols for Illumina Sequencing. *Current Protocols in Human Genetics*, *79*(1), 18.2.1-18.2.42. <https://doi.org/10.1002/0471142905.hg1802s79>

Danecek, P., Auton, A., Abecasis, G., Albers, C. A., Banks, E., DePristo, M. A., Handsaker, R. E., Lunter, G., Marth, G. T., Sherry, S. T., McVean, G., Durbin, R., & 1000 Genomes Project Analysis Group. (2011). The variant call format and VCFtools. *Bioinformatics*, *27*(15), 2156–2158. https://doi.org/10.1093/bioinformatics/btr330

Delussu, F., and Wilkens, B. 2001. Analisi dei resti ossei della tonnara di Pedras de Fogu. Archeologia Postmedievale, 5: 214.

Edwards, S. M. (2017). lemon: Freshing Up your'ggplot2'Plots

GATK. (2016). *GATK Hands ­On Tutorial: Variant Discovery with GATK*. https://qcb.ucla.edu/wp-content/uploads/sites/14/2016/03/GATK_Discovery_Tutorial-Worksheet-AUS2016.pdf

Ginolhac, A., Rasmussen, M., Gilbert, M. T. P., Willerslev, E., & Orlando, L. (2011). mapDamage: Testing for damage patterns in ancient DNA sequences. *Bioinformatics*, *27*(15), 2153–2155. <https://doi.org/10.1093/bioinformatics/btr347>

Gutiérrez-Mas, J. M., Gómez Fernández, V., García López, S., Morales González, J. A., Ibáñez Ageitos, J. M., and Others. 2016. Comparative analysis of the deposits left by the tsunami that followed to the Lisbon earthquake (1755 ad), on the Castilnovo beach and the old tuna factory of la Chança (Conil de la Frontera, SW Spain). Revista de la Sociedad Geológica de España, 29: 21–33.

Gondek, A. T., Boessenkool, S., & Star, B. (2018). A stainless-steel mortar, pestle and sleeve design for the efficient fragmentation of ancient bone. *BioTechniques*, *64*(6), 266–269. <https://doi.org/10.2144/btn-2018-0008>

Jombart, T. (2008). adegenet: A R package for the multivariate analysis of genetic markers. *Bioinformatics*, *24*(11), 1403–1405. <https://doi.org/10.1093/bioinformatics/btn129>

Jónsson, H., Ginolhac, A., Schubert, M., Johnson, P. L. F., & Orlando, L. (2013). mapDamage2.0: Fast approximate Bayesian estimates of ancient DNA damage parameters. *Bioinformatics*, *29*(13), 1682–1684. <https://doi.org/10.1093/bioinformatics/btt193>

Knaus, B. J., & Grünwald, N. J. (2017). vcfr: A package to manipulate and visualize variant call format data in R. *Molecular Ecology Resources*, *17*(1), 44–53. https://doi.org/10.1111/1755-0998.12549

Li, H., & Durbin, R. (2009a). Fast and accurate short read alignment with Burrows–Wheeler transform. *Bioinformatics*, *25*(14), 1754–1760. https://doi.org/10.1093/bioinformatics/btp324

Li, H., Handsaker, B., Wysoker, A., Fennell, T., Ruan, J., Homer, N., Marth, G., Abecasis, G., Durbin, R., & 1000 Genome Project Data Processing Subgroup. (2009b). The Sequence Alignment/Map format and SAMtools. *Bioinformatics*, *25*(16), 2078–2079. https://doi.org/10.1093/bioinformatics/btp352

Nguyen, L.-T., Schmidt, H. A., von Haeseler, A., & Minh, B. Q. (2015). IQ-TREE: A Fast and Effective Stochastic Algorithm for Estimating Maximum-Likelihood Phylogenies. *Molecular Biology and Evolution*, *32*(1), 268–274. <https://doi.org/10.1093/molbev/msu300>

Onar, V., Pazvant, G., and Armutak, A. (2008). Radiocarbon dating results of the animal remains uncovered at Yenikapı Excavations. In Istanbul Archaeological Museums, Proceedings of the 1st Symposium on Marmaray-Metro Salvage Excavations, 249–256.

Paradis, E., Claude, J., & Strimmer, K. (2004). APE: Analyses of Phylogenetics and Evolution in R language. *Bioinformatics*, *20*(2), 289–290. https://doi.org/10.1093/bioinformatics/btg412

Paradis, E. (2010). pegas: An R package for population genetics with an integrated–modular approach. *Bioinformatics*, *26*(3), 419–420. <https://doi.org/10.1093/bioinformatics/btp696>

G. Piquès, “Étude de l’ichtyofaune : déchets de préparation de thons et autres restes de poissons” in Provence-Alpes-Côte d’Azur, Bouches-Du-Rhône, Marseille, 22 Rue Jean-François Leca, Le Castel, Avant Le Port de La Joliette, A. Richier, Ed. (INRAP Rapport final d’opération, 2020), p. 448

Rambaut, A., Drummond, A. J., Xie, D., Baele, G., & Suchard, M. A. (2018). Posterior Summarization in Bayesian Phylogenetics Using Tracer 1.7. *Systematic Biology*, *67*(5), 901–904. https://doi.org/10.1093/sysbio/syy032

Schubert, M., Lindgreen, S., & Orlando, L. (2016). AdapterRemoval v2: Rapid adapter trimming, identification, and read merging. *BMC Research Notes*, *9*(1), 88. <https://doi.org/10.1186/s13104-016-1900-2>

Schroeder, H., Ávila-Arcos, M. C., Malaspinas, A.-S., Poznik, G. D., Sandoval-Velasco, M., Carpenter, M. L., Moreno-Mayar, J. V., Sikora, M., Johnson, P. L. F., Allentoft, M. E., Samaniego, J. A., Haviser, J. B., Dee, M. W., Stafford, T. W., Salas, A., Orlando, L., Willerslev, E., Bustamante, C. D., & Gilbert, M. T. P. (2015). Genome-wide ancestry of 17th-century enslaved Africans from the Caribbean. *Proceedings of the National Academy of Sciences*, *112*(12), 3669–3673. https://doi.org/10.1073/pnas.1421784112

Wickham, H., Averick, M., Bryan, J., Chang, W., McGowan, L. D., François, R., Grolemund, G., Hayes, A., Henry, L., Hester, J., Kuhn, M., Pedersen, T. L., Miller, E., Bache, S. M., Müller, K., Ooms, J., Robinson, D., Seidel, D. P., Spinu, V., … Yutani, H. (2019). Welcome to the Tidyverse. *Journal of Open Source Software*, *4*(43), 1686. https://doi.org/10.21105/joss.01686

Section 2: Supplementary Tables

*Table S1: Sample locations, laboratory protocols and dsDNA concentration for the ancient Atlantic bluefin samples from Norway. All samples were taken from bone tissue (vertebrae) and all samples were dated by context to be from 3000 BCE. More information about the sites can be found in the archaeological reports (Nielsen, 2020a, 2020b, 2020c).*

| **Sample-ID** | **Coordinates** | **Extraction protocol** | **Qubit concentration** | **Library protocol** |
| --- | --- | --- | --- | --- |
| aTUNn02-Norway-3000BCE | N 58.28 E 8.50 | DD | 1.24 | M&K DS |
| aTUNn03-Norway-3000BCE | N 58.28 E 8.50 | DD | 0.48 | M&K DS |
| aTUNn04-Norway-3000BCE | N 58.28 E 8.50 | DD | 1.27 | M&K DS |
| aTUNn05-Norway-3000BCE | N 58.28 E 8.50 | DD | 1.02 | M&K DS |
| aTUNn06-Norway-3000BCE | N 58.28 E 8.50 | DD | 2.7 | M&K DS |
| aTUNn07-Norway-3000BCE | N 58.28 E 8.50 | bleDD | n.a. | M&K DS |
| aTUNn08-Norway-3000BCE | N 58.28 E 8.50 | bleDD | n.a. | M&K DS |
| aTUNn09-Norway-3000BCE | N 58.28 E 8.50 | bleDD | n.a. | M&K DS |
| aTUNn10-Norway-3000BCE | N 58.28 E 8.50 | bleDD | n.a. | M&K DS |
| aTUNn11-Norway-3000BCE | N 58.28 E 8.50 | bleDD | n.a. | M&K DS |
| aTUNn12-Norway-3000BCE | N 58.28 E 8.50 | bleDD | n.a. | M&K DS |
| aTUNn13-Norway-3000BCE | N 58.28 E 8.50 | bleDD | n.a. | M&K DS |
| aTUNn14-Norway-3000BCE | N 58.28 E 8.50 | bleDD | n.a. | M&K DS |
| aTUNn15-Norway-3000BCE | N 58.28 E 8.50 | bleDD | n.a. | M&K DS |
| aTUNn16-Norway-3000BCE | N 58.28 E 8.50 | bleDD | n.a. | M&K DS |
| aTUNn17-Norway-3000BCE | N 58.28 E 8.50 | bleDD | 1.28 | SC SS |
| aTUNn18-Norway-3000BCE | N 58.28 E 8.50 | bleDD | 13.3 | SC SS |
| aTUNn19-Norway-3000BCE | N 58.28 E 8.50 | bleDD | 5.6 | SC SS |
| aTUNn20-Norway-3000BCE | N 58.28 E 8.50 | bleDD | 1.6 | SC SS |
| aTUNn21-Norway-3000BCE | N 58.28 E 8.50 | bleDD | 2.8 | SC SS |
| aTUNn22-Norway-3000BCE | N 58.28 E 8.50 | bleDD | 5.56 | SC SS |
| aTUNn23-Norway-3000BCE | N 58.28 E 8.50 | bleDD | 19.6 | SC SS |
| aTUNn24-Norway-3000BCE | N 58.28 E 8.50 | bleDD | 3.54 | SC SS |
| aTUNn25-Norway-3000BCE | N 58.28 E 8.50 | bleDD | 11 | SC SS |
| aTUNn26-Norway-3000BCE | N 58.28 E 8.50 | bleDD | 6.94 | SC SS |
| aTUNn27-Norway-3000BCE | N 58.28 E 8.50 | bleDD | 2.8 | SC SS |
| aTUNn28-Norway-3000BCE | N 58.28 E 8.50 | bleDD | 10.9 | SC SS |
| aTUNn29-Norway-3000BCE | N 58.28 E 8.50 | bleDD | 89.6 | SC SS |
| aTUNn30-Norway-3000BCE | N 58.28 E 8.50 | bleDD | 1.87 | SC SS |
| aTUNn31-Norway-3000BCE | N 58.28 E 8.50 | bleDD | 19.9 | SC SS |
| aTUNn32-Norway-3000BCE | N 58.28 E 8.50 | bleDD | 26 | SC SS |
| aTUNn33-Norway-3000BCE | N 58.28 E 8.50 | bleDD | 1.9 | SC SS |
| aTUNn34-Norway-3000BCE | N 58.28 E 8.50 | bleDD | 36 | SC SS |
| aTUNn35-Norway-3000BCE | N 58.28 E 8.50 | bleDD | 78.8 | SC SS |
| aTUNn36-Norway-3000BCE | N 58.28 E 8.50 | bleDD | 25.2 | SC SS |
| aTUNn37-Norway-3000BCE | N 58.28 E 8.50 | bleDD | 3.18 | SC SS |
| aTUNn38-Norway-3000BCE | N 58.28 E 8.50 | bleDD | 3.06 | SC SS |
| aTUNn39-Norway-3000BCE | N 58.28 E 8.50 | bleDD | 1.4 | SC SS |
| DD: Double digestion. Extraction protocol adapted from Damgaard et al. 2015.  bleDD: Bleach and double digestion. Extraction protocol adapted from Boessenkool et al. 2017.  M&K DS: Double stranded libraries. Library protocol adapted from Meyer and Kircher 2010 with modifications from Schroeder et al. 2015.  SC SS: Santa Cruz Reaction single-stranded library protocol from Kapp, Green, and Shapiro 2021. | | | | |

*Table S2: Sample locations, date sampled and for the ancient Atlantic bluefin samples from the Mediterranean. All samples were taken from bone tissue (vertebrae). Samples were dated by context at the archaeological sites. More information about the sites can be found in Andrews et al. (2023b).*

| **Sample-ID** | **Coordinates** | **Location** | **Date of origin** |
| --- | --- | --- | --- |
| aTUNm01-Sicily-900-1200CE | N 38.11 E 13.37 | Palermo | 900-1000 CE |
| aTUNm02-Sicily-900-1200CE | N 38.11 E 13.37 | Palermo | 900-1000 CE |
| aTUNm03-Sicily-900-1200CE | N 38.11 E 13.37 | Palermo | 900-1000 CE |
| aTUNm04-Sicily-900-1200CE | N 38.11 E 13.37 | Palermo | 900-1000 CE |
| aTUNm05-Sicily-900-1200CE | N 38.11 E 13.37 | Palermo | 900-1000 CE |
| aTUNm06-Sicily-900-1200CE | N 38.11 E 13.37 | Palermo | 900-1000 CE |
| aTUNm07-Sicily-900-1200CE | N 37.65 E 12.59 | Mazara del Vallo | 1200 CE |
| aTUNm08-Sicily-900-1200CE | N 37.65 E 12.59 | Mazara del Vallo | 1200 CE |
| aTUNm09-Sicily-900-1200CE | N 37.65 E 12.59 | Mazara del Vallo | 1200 CE |
| aTUNm10-Sicily-900-1200CE | N 37.65 E 12.59 | Mazara del Vallo | 1200 CE |
| aTUNm11-Sicily-900-1200CE | N 38.11 E 13.36 | Palermo | 900-1000 CE |
| aTUNm12-Sicily-900-1200CE | N 38.11 E 13.36 | Palermo | 900-1000 CE |
| aTUNm13-Zliten-1925CE | N 33.25 E 14.66 | Zliten | 1925 CE |
| aTUNm14-Zliten-1925CE | N 33.25 E 14.66 | Zliten | 1925 CE |
| aTUNm15-Zliten-1925CE | N 33.25 E 14.66 | Zliten | 1925 CE |
| aTUNm16-Zliten-1925CE | N 33.25 E 14.66 | Zliten | 1925 CE |
| aTUNm17-Zliten-1925CE | N 33.25 E 14.66 | Zliten | 1925 CE |
| aTUNm18-Zliten-1925CE | N 33.25 E 14.66 | Zliten | 1925 CE |
| aTUNm19-Zliten-1925CE | N 33.25 E 14.66 | Zliten | 1925 CE |
| aTUNm20-Zliten-1925CE | N 33.25 E 14.66 | Zliten | 1925 CE |
| aTUNm21-Zliten-1925CE | N 33.25 E 14.66 | Zliten | 1925 CE |
| aTUNm22-Zliten-1925CE | N 33.25 E 14.66 | Zliten | 1925 CE |
| aTUNm23-Zliten-1925CE | N 33.25 E 14.66 | Zliten | 1925 CE |
| aTUNm24-Zliten-1925CE | N 33.25 E 14.66 | Zliten | 1925 CE |
| aTUNm25-Zliten-1925CE | N 33.25 E 14.66 | Zliten | 1925 CE |
| aTUNm26-Zliten-1925CE | N 33.25 E 14.66 | Zliten | 1925 CE |
| aTUNm27-Zliten-1925CE | N 33.25 E 14.66 | Zliten | 1925 CE |
| aTUNm28-Zliten-1925CE | N 33.25 E 14.66 | Zliten | 1925 CE |
| aTUNm29-Zliten-1925CE | N 33.25 E 14.66 | Zliten | 1925 CE |
| aTUNm30-Zliten-1925CE | N 33.25 E 14.66 | Zliten | 1925 CE |
| aTUNm31-Zliten-1925CE | N 33.25 E 14.66 | Zliten | 1925 CE |
| aTUNm32-Zliten-1925CE | N 33.25 E 14.66 | Zliten | 1925 CE |
| aTUNm33-Zliten-1925CE | N 33.25 E 14.66 | Zliten | 1925 CE |
| aTUNm34-Zliten-1925CE | N 33.25 E 14.66 | Zliten | 1925 CE |
| aTUNm35-Istanbul-1941CE | N 41.01 E 28.95 | Istanbul | 1941 CE |
| aTUNm36-Istanbul-1941CE | N 41.01 E 28.95 | Istanbul | 1941 CE |
| aTUNm37-Istanbul-800-1200CE | N 41.01 E 28.95 | Istanbul | 800-1200 CE |
| aTUNm38-Istanbul-800-1200CE | N 41.01 E 28.95 | Istanbul | 800-1200 CE |
| aTUNm39-Istanbul-800-1200CE | N 41.01 E 28.95 | Istanbul | 800-1200 CE |
| aTUNm40-Istanbul-800-1200CE | N 41.01 E 28.95 | Istanbul | 800-1200 CE |
| aTUNm41-Istanbul-800-1200CE | N 41.01 E 28.95 | Istanbul | 800-1200 CE |
| aTUNm42-Istanbul-800-1200CE | N 41.01 E 28.95 | Istanbul | 800-1200 CE |
| aTUNm43-Istanbul-800-1200CE | N 41.01 E 28.95 | Istanbul | 800-1200 CE |
| aTUNm44-Istanbul-800-1200CE | N 41.01 E 28.95 | Istanbul | 800-1200 CE |
| aTUNm45-Istanbul-800-1200CE | N 41.01 E 28.95 | Istanbul | 800-1200 CE |
| aTUNm46-Istanbul-800-1200CE | N 41.01 E 28.95 | Istanbul | 800-1200 CE |
| aTUNm47-Istanbul-800-1200CE | N 41.01 E 28.95 | Istanbul | 800-1200 CE |
| aTUNm48-Istanbul-800-1200CE | N 41.01 E 28.95 | Istanbul | 800-1200 CE |
| aTUNm49-Istanbul-800-1200CE | N 41.01 E 28.95 | Istanbul | 800-1200 CE |
| aTUNm50-Istanbul-800-1200CE | N 41.01 E 28.95 | Istanbul | 800-1200 CE |
| aTUNm51-Istanbul-800-1200CE | N 41.01 E 28.95 | Istanbul | 800-1200 CE |
| aTUNm52-Istanbul-800-1200CE | N 41.01 E 28.95 | Istanbul | 800-1200 CE |
| aTUNm53-Istanbul-800-1200CE | N 41.01 E 28.95 | Istanbul | 800-1200 CE |
| aTUNm54-Istanbul-800-1200CE | N 41.01 E 28.95 | Istanbul | 800-1200 CE |
| aTUNm55-Istanbul-800-1200CE | N 41.01 E 28.95 | Istanbul | 800-1200 CE |
| aTUNm56-Istanbul-800-1200CE | N 41.01 E 28.95 | Istanbul | 800-1200 CE |
| aTUNm57-Istanbul-800-1200CE | N 41.01 E 28.95 | Istanbul | 800-1200 CE |
| aTUNm58-Gibraltar-1755CE | N 36.28 W 6.09 | Conil | 1755 CE |
| aTUNm59-Gibraltar-1755CE | N 36.28 W 6.09 | Conil | 1755 CE |
| aTUNm60-Gibraltar-1755CE | N 36.28 W 6.09 | Conil | 1755 CE |
| aTUNm61-Gibraltar-1755CE | N 36.28 W 6.09 | Conil | 1755 CE |
| aTUNm62-Gibraltar-1755CE | N 36.28 W 6.09 | Conil | 1755 CE |
| aTUNm63-Gibraltar-1755CE | N 36.28 W 6.09 | Conil | 1755 CE |
| aTUNm64-Gibraltar-1755CE | N 36.28 W 6.09 | Conil | 1755 CE |
| aTUNm65-Marseille-1800CE | N 43.30 E 5.37 | Marseille | 1800 CE |
| aTUNm66-Marseille-1800CE | N 43.30 E 5.37 | Marseille | 1800 CE |
| aTUNm67-Marseille-1800CE | N 43.30 E 5.37 | Marseille | 1800 CE |
| aTUNm68-Marseille-1800CE | N 43.30 E 5.37 | Marseille | 1800 CE |
| aTUNm69-Marseille-1800CE | N 43.30 E 5.37 | Marseille | 1800 CE |
| aTUNm70-Marseille-1800CE | N 43.30 E 5.37 | Marseille | 1800 CE |
| aTUNm71-Marseille-1800CE | N 43.30 E 5.37 | Marseille | 1800 CE |
| aTUNm72-Marseille-1800CE | N 43.30 E 5.37 | Marseille | 1800 CE |
| aTUNm73-Gibraltar-100CE | N 36.53 W 6.30 | Cadiz | 100 CE |
| aTUNm74-Gibraltar-100CE | N 36.53 W 6.30 | Cadiz | 100 CE |
| aTUNm75-Gibraltar-100CE | N 36.53 W 6.30 | Cadiz | 100 CE |
| aTUNm76-Gibraltar-100CE | N 36.53 W 6.30 | Cadiz | 100 CE |
| aTUNm77-Gibraltar-100CE | N 36.53 W 6.30 | Cadiz | 100 CE |
| aTUNm78-Sardinia-1500-1700CE | N 40.86 E 8.62 | Sassari | 1500-1700 CE |
| aTUNm79-Sardinia-1500-1700CE | N 40.86 E 8.62 | Sassari | 1500-1700 CE |
| aTUNm80-Sardinia-1500-1700CE | N 40.86 E 8.62 | Sassari | 1500-1700 CE |
| aTUNm81-Sardinia-1500-1700CE | N 40.86 E 8.62 | Sassari | 1500-1700 CE |
| aTUNm82-Sardinia-1500-1700CE | N 40.86 E 8.62 | Sassari | 1500-1700 CE |
| aTUNm83-Sardinia-1500-1700CE | N 40.86 E 8.62 | Sassari | 1500-1700 CE |
| aTUNm84-Sardinia-1500-1700CE | N 40.86 E 8.62 | Sassari | 1500-1700 CE |
| aTUNm85-Sardinia-1500-1700CE | N 40.86 E 8.62 | Sassari | 1500-1700 CE |
| aTUNm86-Sardinia-1500-1700CE | N 40.86 E 8.62 | Sassari | 1500-1700 CE |
| aTUNm87-Sardinia-1500-1700CE | N 40.86 E 8.62 | Sassari | 1500-1700 CE |
| aTUNm88-Sardinia-1500-1700CE | N 40.86 E 8.62 | Sassari | 1500-1700 CE |
| aTUNm89-Sardinia-1500-1700CE | N 40.86 E 8.62 | Sassari | 1500-1700 CE |
| aTUNm90-Sardinia-1500-1700CE | N 40.86 E 8.62 | Sassari | 1500-1700 CE |
| aTUNm91-Sardinia-1500-1700CE | N 40.86 E 8.62 | Sassari | 1500-1700 CE |
| aTUNm92-Sardinia-1500-1700CE | N 40.86 E 8.62 | Sassari | 1500-1700 CE |

*Table S3: Sample locations, lifestage, tissue, date sampled and dsDNA concentration for the modern Atlantic bluefin samples from Norway.*

| **Sample-ID** | **Coordinates** | **Lifestage and Estimated TW (kg)** | **Tissue** | **Year sampled** | **Qubit concentration (ng/µl)** |
| --- | --- | --- | --- | --- | --- |
| mTUNn01-NOR | N 63.65 E 7.95 | Adult, 151.2 | Powdered muscle | 2018 | 5.5 |
| mTUNn02-NOR | N 63.65 E 7.95 | Adult, 218.6 | Powdered muscle | 2018 | 20.6 |
| mTUNn03-NOR | N 63.65 E 7.95 | Adult, 219.2 | Powdered muscle | 2018 | 20.0 |
| mTUNn04-NOR | N 63.65 E 7.95 | Adult, 225.5 | Powdered muscle | 2018 | 4.5 |
| mTUNn05-NOR | N 63.65 E 7.95 | Adult, 217.4 | Powdered muscle | 2018 | 9.2 |
| mTUNn06-NOR | N 63.65 E 7.95 | Adult, 221.1 | Powdered muscle | 2018 | 5.2 |
| mTUNn07-NOR | N 63.65 E 7.95 | Adult, 228.7 | Powdered muscle | 2018 | 10.2 |
| mTUNn08-NOR | N 63.65 E 7.95 | Adult, 227.4 | Powdered muscle | 2018 | 5.1 |
| mTUNn09-NOR | N 63.65 E 7.95 | Adult, 240.7 | Powdered muscle | 2018 | 11.7 |
| mTUNn10-NOR | N 63.65 E 7.95 | Adult, 264.0 | Powdered muscle | 2018 | 6.0 |
| mTUNn11-NOR | N 62.90 E 6.00 | Adult, 215.5 | Fin skin | 2020 | 53.4 |
| mTUNn12-NOR | N 62.90 E 6.00 | Adult, 202.9 | Fin skin | 2020 | 43.8 |
| mTUNn13-NOR | N 62.90 E 6.00 | Adult, 182.7 | Fin skin | 2020 | 31.4 |
| mTUNn14-NOR | N 62.90 E 6.00 | Adult, 313.7 | Fin skin | 2020 | 83.7 |
| mTUNn15-NOR | N 62.90 E 6.00 | Adult, 310.0 | Fin skin | 2020 | 27.3 |
| mTUNn16-NOR | N 62.90 E 6.00 | Adult, 181.4 | Fin skin | 2020 | 41.8 |
| mTUNn17-NOR | N 62.90 E 6.00 | Adult, 225.5 | Fin skin | 2020 | 13.4 |
| mTUNn18-NOR | N 62.90 E 6.00 | Adult, 170.1 | Fin skin | 2020 | 44.0 |
| mTUNn19-NOR | N 62.90 E 6.00 | Adult, 194.0 | Fin skin | 2020 | 91.9 |
| mTUNn20-NOR | N 62.90 E 6.00 | Adult, 220.5 | Fin skin | 2020 | 28.5 |
| mTUNn21-NOR | N 62.90 E 6.00 | Adult, 206.6 | Fin skin | 2020 | 81.3 |
| mTUNn22-NOR | N 62.90 E 6.00 | Adult, 250.7 | Fin skin | 2020 | 58.8 |
| mTUNn23-NOR | N 62.90 E 6.00 | Adult, 190.3 | Fin skin | 2020 | 19.1 |
| mTUNn24-NOR | N 62.90 E 6.00 | Adult, 270.9 | Fin skin | 2020 | 23.6 |
| mTUNn25-NOR | N 62.90 E 6.00 | Adult, 187.7 | Fin skin | 2020 | 73.4 |
| mTUNn26-NOR | N 62.90 E 6.00 | Adult, 186.5 | Fin skin | 2020 | 70.5 |
| mTUNn27-NOR | N 62.90 E 6.00 | Adult, 264.6 | Fin skin | 2020 | 38.7 |
| mTUNn28-NOR | N 62.90 E 6.00 | Adult, 241.9 | Fin skin | 2020 | 41.2 |
| mTUNn29-NOR | N 62.90 E 6.00 | Adult, 296.1 | Fin skin | 2020 | 48.3 |
| mTUNn30-NOR | N 62.90 E 6.00 | Adult, 260.8 | Fin skin | 2020 | 32.0 |
| mTUNn31-NOR | N 62.90 E 6.00 | Adult, 214.2 | Fin skin | 2020 | 21.8 |
| mTUNn32-NOR | N 62.90 E 6.00 | Adult, 170.1 | Fin skin | 2020 | 43.2 |
| mTUNn33-NOR | N 62.90 E 6.00 | Adult, 205.4 | Fin skin | 2020 | 36.1 |
| mTUNn34-NOR | N 62.90 E 6.00 | Adult, 181.4 | Fin skin | 2020 | 36.3 |
| mTUNn35-NOR | N 62.90 E 6.00 | Adult, 182.7 | Fin skin | 2020 | 43.8 |
| mTUNn36-NOR | N 62.90 E 6.00 | Adult, 202.9 | Fin skin | 2020 | 32.6 |
| mTUNn37-NOR | N 62.90 E 6.00 | Adult, 189.0 | Fin skin | 2020 | 66.5 |
| mTUNn38-NOR | N 62.90 E 6.00 | Adult, 204.1 | Fin skin | 2020 | 20.4 |

*Table S4: Sample locations, lifestage, tissue and date sampled for the modern Atlantic bluefin samples from the Mediterranean and the Gulf of Mexico.*

| **Sample-ID** | **Coordinates** | **Location** | **Lifestage** | **Year sampled** |
| --- | --- | --- | --- | --- |
| mTUNm01-EMED | N 35.51 E 33.42 | Cyprus | YoY | 2013 |
| mTUNm02-EMED | N 35.51 E 33.42 | Cyprus | YoY | 2013 |
| mTUNm03-EMED | N 35.51 E 33.42 | Cyprus | YoY | 2013 |
| mTUNm04-EMED | N 35.51 E 33.42 | Cyprus | YoY | 2013 |
| mTUNm05-EMED | N 35.51 E 33.42 | Cyprus | YoY | 2013 |
| mTUNm06-EMED | N 35.51 E 33.42 | Cyprus | YoY | 2013 |
| mTUNm07-EMED | N 35.51 E 33.42 | Cyprus | YoY | 2013 |
| mTUNm08-EMED | N 35.51 E 33.42 | Cyprus | YoY | 2013 |
| mTUNm09-EMED | N 35.51 E 33.42 | Cyprus | YoY | 2013 |
| mTUNm10-EMED | N 35.51 E 33.42 | Cyprus | YoY | 2013 |
| mTUNm11-WMED | N 39.27 E 2.07 | Palma | YoY | 2013 |
| mTUNm12-WMED | N 39.27 E 2.07 | Palma | YoY | 2013 |
| mTUNm13-WMED | N 39.27 E 2.07 | Palma | YoY | 2013 |
| mTUNm14-WMED | N 39.27 E 2.07 | Palma | YoY | 2013 |
| mTUNm15-WMED | N 39.27 E 2.07 | Palma | YoY | 2013 |
| mTUNm16-WMED | N 39.27 E 2.07 | Palma | YoY | 2013 |
| mTUNm17-WMED | N 39.27 E 2.07 | Palma | YoY | 2013 |
| mTUNm18-WMED | N 39.27 E 2.07 | Palma | YoY | 2013 |
| mTUNm19-WMED | N 39.27 E 2.07 | Palma | YoY | 2013 |
| mTUNm20-WMED | N 39.27 E 2.07 | Palma | YoY | 2013 |
| mTUNm31-CMED | N 36.93 E 13.18 | Sicily | YoY | 2013 |
| mTUNm32-CMED | N 36.93 E 13.18 | Sicily | YoY | 2013 |
| mTUNm33-CMED | N 36.93 E 13.18 | Sicily | YoY | 2013 |
| mTUNm34-CMED | N 36.93 E 13.18 | Sicily | YoY | 2013 |
| mTUNm35-CMED | N 36.93 E 13.18 | Sicily | YoY | 2013 |
| mTUNm36-CMED | N 36.93 E 13.18 | Sicily | YoY | 2013 |
| mTUNm37-CMED | N 36.93 E 13.18 | Sicily | YoY | 2013 |
| mTUNm38-CMED | N 36.93 E 13.18 | Sicily | YoY | 2013 |
| mTUNm39-CMED | N 36.93 E 13.18 | Sicily | YoY | 2013 |
| mTUNm40-CMED | N 36.93 E 13.18 | Sicily | YoY | 2013 |
| mTUNm41-GOM | N 26.12 W 87.78 | Gulf of Mexico | larvae | 2017 |
| mTUNm42-GOM | N 25.84 W 88.13 | Gulf of Mexico | larvae | 2017 |
| mTUNm43-GOM | N 28.33 W 87.25 | Gulf of Mexico | larvae | 2018 |
| mTUNm44-GOM | N 28.33 W 87.25 | Gulf of Mexico | larvae | 2018 |
| mTUNm45-GOM | N 28.33 W 87.25 | Gulf of Mexico | larvae | 2018 |
| mTUNm46-GOM | N 26.53 W 93.58 | Gulf of Mexico | larvae | 2014 |
| mTUNm47-GOM | N 26.53 W 93.58 | Gulf of Mexico | larvae | 2014 |
| mTUNm48-GOM | N 27.04 W 93.00 | Gulf of Mexico | larvae | 2014 |
| mTUNm49-GOM | N 28.00 W 87.76 | Gulf of Mexico | larvae | 2014 |
| mTUNm50-GOM | N 28.00 W 87.76 | Gulf of Mexico | larvae | 2014 |

*Table S5: Sample locations, lifestage, tissue and date sampled for the modern albacore samples from the Bay of Biscay.*

| **Sample-ID** | **Coordinates** | **Location** | **Lifestage** | **Year sampled** |
| --- | --- | --- | --- | --- |
| ALB-TUN-01 | N 44.46 W 3.12 | Bay of Biscay | Juvenile (5-15 kg) | 2010 |
| ALB-TUN-02 | N 44.46 W 3.12 | Bay of Biscay | Juvenile (5-15 kg) | 2010 |
| ALB-TUN-03 | N 44.46 W 3.12 | Bay of Biscay | Juvenile (5-15 kg) | 2010 |
| ALB-TUN-04 | N 44.46 W 3.12 | Bay of Biscay | Juvenile (5-15 kg) | 2010 |
| ALB-TUN-05 | N 44.46 W 3.12 | Bay of Biscay | Juvenile (5-15 kg) | 2010 |
| ALB-TUN-06 | N 44.46 W 3.12 | Bay of Biscay | Juvenile (5-15 kg) | 2010 |

*Table S6: Pacific bluefin whole genome raw sequences downloaded from the DDBJ database (Kodama et al., 2012).*

| **Original sample-ID** | **Sample-ID** | **DDBJ identifier** | **Filename** |
| --- | --- | --- | --- |
| PAC-DRR177383 | PBFT-TUN-01 | DRA008331 | DRR177383_1.fastq.bz2 |
|  |  |  | DRR177383_2.fastq.bz2 |
| PAC-DRR177395 | PBFT-TUN-02 | DRA008331 | DRR177395_1.fastq.bz2 |
|  |  |  | DRR177395_2.fastq.bz2 |
| PAC-DRR177400 | PBFT-TUN-03 | DRA008331 | DRR177400_1.fastq.bz2 |
|  |  |  | DRR177400_2.fastq.bz2 |
| PAC-DRR177401 | PBFT-TUN-04 | DRA008331 | DRR177401_1.fastq.bz2 |
|  |  |  | DRR177401_2.fastq.bz2 |
| PAC-DRR177402 | PBFT-TUN-05 | DRA008331 | DRR177402_1.fastq.bz2 |
|  |  |  | DRR177402_2.fastq.bz2 |
| PAC-DRR177403 | PBFT-TUN-06 | DRA008331 | DRR177403_1.fastq.bz2 |
|  |  |  | DRR177403_2.fastq.bz2 |
| PAC-DRR177404 | PBFT-TUN-07 | DRA008331 | DRR177404_1.fastq.bz2 |
|  |  |  | DRR177404_2.fastq.bz2 |
| PAC-DRR177405 | PBFT-TUN-08 | DRA008331 | DRR177405_1.fastq.bz2 |
|  |  |  | DRR177405_2.fastq.bz2 |
| PAC-DRR177406 | PBFT-TUN-09 | DRA008331 | DRR177406_1.fastq.bz2 |
|  |  |  | DRR177406_2.fastq.bz2 |
| URL: https://ddbj.nig.ac.jp/public/ddbj_database/dra/fastq/DRA008/DRA008331/DRX167946/ | | | |

*Table S7: Summary statistics from Paleomix for the ancient Atlantic bluefin specimens from Norway. The endogenous content is calculated from the alignment to the Atlantic bluefin nuclear reference genome. Samples that were removed from further analyses are marked with a star (*).*

| **Sample-ID** | **Reads (millions)** | **Endogenous  DNA (fraction)** | **Mitochondrial coverage** | **Mean fragment length (bp)** |
| --- | --- | --- | --- | --- |
| aTUNn02-Norway-3000BCE | 32 | 0.06 | 7 | 74 |
| aTUNn03-Norway-3000BCE | 42 | 0.15 | 15 | 64 |
| aTUNn04-Norway-3000BCE | 21 | 0.16 | 8 | 62 |
| aTUNn05-Norway-3000BCE | 120 | 0.51 | 164 | 64 |
| aTUNn06-Norway-3000BCE | 528 | 0.60 | 1546 | 78 |
| aTUNn07-Norway-3000BCE | 5 | 0.26 | 5 | 77 |
| aTUNn08-Norway-3000BCE | 16 | 0.14 | 6 | 66 |
| aTUNn09-Norway-3000BCE | 17 | 0.50 | 17 | 63 |
| aTUNn10-Norway-3000BCE * | 3 | 0.22 | 2 | 80 |
| aTUNn11-Norway-3000BCE | 13 | 0.48 | 14 | 66 |
| aTUNn12-Norway-3000BCE * | 20 | 0.46 | 18 | 64 |
| aTUNn13-Norway-3000BCE | 26 | 0.54 | 29 | 67 |
| aTUNn14-Norway-3000BCE * | 8 | 0.33 | 7 | 66 |
| aTUNn15-Norway-3000BCE | 10 | 0.17 | 3 | 70 |
| aTUNn16-Norway-3000BCE | 2 | 0.50 | 2 | 66 |
| aTUNn17-Norway-3000BCE | 6 | 0.37 | 11 | 100 |
| aTUNn18-Norway-3000BCE * | 6 | 0.03 | 1 | 79 |
| aTUNn19-Norway-3000BCE | 88 | 0.20 | 115 | 109 |
| aTUNn20-Norway-3000BCE | 19 | 0.32 | 38 | 101 |
| aTUNn21-Norway-3000BCE | 9 | 0.38 | 23 | 86 |
| aTUNn22-Norway-3000BCE | 14 | 0.37 | 34 | 101 |
| aTUNn23-Norway-3000BCE | 12 | 0.03 | 3 | 109 |
| aTUNn24-Norway-3000BCE | 153 | 0.64 | 318 | 91 |
| aTUNn25-Norway-3000BCE * | 14 | 0.23 | 23 | 81 |
| aTUNn26-Norway-3000BCE * | 11 | 0.17 | 10 | 95 |
| aTUNn27-Norway-3000BCE * | 16 | 0.34 | 38 | 85 |
| aTUNn28-Norway-3000BCE * | 15 | 0.04 | 5 | 97 |
| aTUNn29-Norway-3000BCE * | 13 | 0.09 | 11 | 106 |
| aTUNn30-Norway-3000BCE | 11 | 0.18 | 4 | 113 |
| aTUNn31-Norway-3000BCE | 15 | 0.12 | 13 | 120 |
| aTUNn32-Norway-3000BCE * | 15 | 0.14 | 12 | 119 |
| aTUNn33-Norway-3000BCE * | 12 | 0.13 | 12 | 111 |
| aTUNn34-Norway-3000BCE * | 29 | 0.00 | 0 | 122 |
| aTUNn35-Norway-3000BCE | 14 | 0.19 | 15 | 116 |
| aTUNn36-Norway-3000BCE * | 14 | 0.03 | 4 | 107 |
| aTUNn37-Norway-3000BCE | 344 | 0.64 | 740 | 77 |
| aTUNn38-Norway-3000BCE * | 25 | 0.47 | 32 | 90 |
| aTUNn39-Norway-3000BCE | 9 | 0.15 | 3 | 121 |

*Table S8: Summary statistics from Paleomix for the ancient Atlantic bluefin specimens from the Mediterranean. The endogenous content is calculated from the alignment to the Atlantic bluefin nuclear reference genome. Samples that were removed from further analyses are marked with a star (*).*

| **Sample-ID** | **Reads**  **(millions)** | **Endogenous DNA (fraction)** | **Mitochondrial**  **coverage** | **Mean fragment length (bp)** |
| --- | --- | --- | --- | --- |
| aTUNm01-Sicily-900-1200CE | 16 | 0.07 | 2 | 83 |
| aTUNm02-Sicily-900-1200CE | 5 | 0.17 | 5 | 79 |
| aTUNm03-Sicily-900-1200CE * | 8 | 0.19 | 5 | 68 |
| aTUNm04-Sicily-900-1200CE * | 5 | 0.17 | 3 | 86 |
| aTUNm05-Sicily-900-1200CE | 18 | 0.12 | 10 | 91 |
| aTUNm06-Sicily-900-1200CE | 6 | 0.21 | 6 | 92 |
| aTUNm07-Sicily-900-1200CE | 6 | 0.52 | 16 | 86 |
| aTUNm08-Sicily-900-1200CE | 5 | 0.22 | 4 | 103 |
| aTUNm09-Sicily-900-1200CE | 8 | 0.07 | 2 | 93 |
| aTUNm10-Sicily-900-1200CE | 16 | 0.58 | 73 | 73 |
| aTUNm11-Sicily-900-1200CE | 7 | 0.59 | 25 | 79 |
| aTUNm12-Sicily-900-1200CE | 7 | 0.12 | 4 | 92 |
| aTUNm13-Zliten-1925CE | 7 | 0.38 | 12 | 70 |
| aTUNm14-Zliten-1925CE | 8 | 0.36 | 15 | 77 |
| aTUNm15-Zliten-1925CE | 7 | 0.06 | 3 | 80 |
| aTUNm16-Zliten-1925CE | 6 | 0.11 | 4 | 68 |
| aTUNm17-Zliten-1925CE | 6 | 0.33 | 5 | 69 |
| aTUNm18-Zliten-1925CE | 8 | 0.40 | 12 | 72 |
| aTUNm19-Zliten-1925CE | 7 | 0.25 | 6 | 75 |
| aTUNm20-Zliten-1925CE | 4 | 0.46 | 7 | 68 |
| aTUNm21-Zliten-1925CE * | 6 | 0.06 | 3 | 70 |
| aTUNm22-Zliten-1925CE | 8 | 0.37 | 13 | 67 |
| aTUNm23-Zliten-1925CE | 7 | 0.15 | 5 | 63 |
| aTUNm24-Zliten-1925CE | 7 | 0.17 | 4 | 66 |
| aTUNm25-Zliten-1925CE | 6 | 0.45 | 9 | 69 |
| aTUNm26-Zliten-1925CE * | 9 | 0.08 | 4 | 72 |
| aTUNm27-Zliten-1925CE | 8 | 0.07 | 5 | 83 |
| aTUNm28-Zliten-1925CE | 9 | 0.25 | 9 | 77 |
| aTUNm29-Zliten-1925CE | 9 | 0.18 | 6 | 77 |
| aTUNm30-Zliten-1925CE | 10 | 0.04 | 3 | 86 |
| aTUNm31-Zliten-1925CE | 7 | 0.49 | 10 | 80 |
| aTUNm32-Zliten-1925CE | 9 | 0.28 | 17 | 83 |
| aTUNm33-Zliten-1925CE | 7 | 0.26 | 6 | 74 |
| aTUNm34-Zliten-1925CE | 6 | 0.10 | 4 | 80 |
| aTUNm35-Istanbul-1941CE | 6 | 0.11 | 1 | 111 |
| aTUNm36-Istanbul-1941CE | 10 | 0.27 | 10 | 72 |
| aTUNm37-Istanbul-800-1200CE | 5 | 0.51 | 22 | 77 |
| aTUNm38-Istanbul-800-1200CE | 5 | 0.47 | 9 | 70 |
| aTUNm39-Istanbul-800-1200CE | 5 | 0.24 | 7 | 82 |
| aTUNm40-Istanbul-800-1200CE * | 4 | 0.45 | 19 | 91 |
| aTUNm41-Istanbul-800-1200CE | 5 | 0.57 | 25 | 76 |
| aTUNm42-Istanbul-800-1200CE | 4 | 0.36 | 7 | 90 |
| aTUNm43-Istanbul-800-1200CE | 4 | 0.32 | 4 | 74 |
| aTUNm44-Istanbul-800-1200CE | 5 | 0.16 | 3 | 76 |
| aTUNm45-Istanbul-800-1200CE | 4 | 0.59 | 16 | 75 |
| aTUNm46-Istanbul-800-1200CE | 4 | 0.53 | 18 | 83 |
| aTUNm47-Istanbul-800-1200CE | 4 | 0.61 | 12 | 77 |
| aTUNm48-Istanbul-800-1200CE * | 4 | 0.09 | 2 | 78 |
| aTUNm49-Istanbul-800-1200CE | 4 | 0.22 | 5 | 79 |
| aTUNm50-Istanbul-800-1200CE | 6 | 0.50 | 12 | 82 |
| aTUNm51-Istanbul-800-1200CE | 10 | 0.46 | 31 | 75 |
| aTUNm52-Istanbul-800-1200CE | 14 | 0.39 | 64 | 78 |
| aTUNm53-Istanbul-800-1200CE | 6 | 0.38 | 8 | 76 |
| aTUNm54-Istanbul-800-1200CE | 9 | 0.33 | 15 | 87 |
| aTUNm55-Istanbul-800-1200CE | 6 | 0.42 | 10 | 77 |
| aTUNm56-Istanbul-800-1200CE | 10 | 0.47 | 17 | 77 |
| aTUNm57-Istanbul-800-1200CE | 4 | 0.55 | 25 | 96 |
| aTUNm58-Gibraltar-1755CE | 10 | 0.06 | 2 | 82 |
| aTUNm59-Gibraltar-1755CE | 8 | 0.10 | 3 | 79 |
| aTUNm60-Gibraltar-1755CE | 6 | 0.10 | 3 | 87 |
| aTUNm61-Gibraltar-1755CE | 8 | 0.08 | 3 | 82 |
| aTUNm62-Gibraltar-1755CE | 6 | 0.16 | 2 | 85 |
| aTUNm63-Gibraltar-1755CE | 5 | 0.10 | 3 | 89 |
| aTUNm64-Gibraltar-1755CE | 14 | 0.06 | 3 | 83 |
| aTUNm65-Marseille-1800CE | 6 | 0.34 | 10 | 72 |
| aTUNm66-Marseille-1800CE | 7 | 0.35 | 69 | 95 |
| aTUNm67-Marseille-1800CE | 9 | 0.22 | 7 | 99 |
| aTUNm68-Marseille-1800CE | 10 | 0.24 | 6 | 82 |
| aTUNm69-Marseille-1800CE | 3 | 0.28 | 8 | 82 |
| aTUNm70-Marseille-1800CE | 6 | 0.25 | 12 | 82 |
| aTUNm71-Marseille-1800CE | 9 | 0.18 | 27 | 92 |
| aTUNm72-Marseille-1800CE | 38 | 0.12 | 14 | 72 |
| aTUNm73-Gibraltar-100CE | 22 | 0.05 | 11 | 92 |
| aTUNm74-Gibraltar-100CE * | 13 | 0.07 | 2 | 83 |
| aTUNm75-Gibraltar-100CE | 21 | 0.13 | 10 | 85 |
| aTUNm76-Gibraltar-100CE | 13 | 0.09 | 5 | 99 |
| aTUNm77-Gibraltar-100CE | 15 | 0.08 | 6 | 82 |
| aTUNm78-Sardinia-1500-1700CE | 5 | 0.39 | 8 | 103 |
| aTUNm79-Sardinia-1500-1700CE | 11 | 0.57 | 29 | 75 |
| aTUNm80-Sardinia-1500-1700CE | 6 | 0.06 | 4 | 105 |
| aTUNm81-Sardinia-1500-1700CE | 6 | 0.39 | 10 | 86 |
| aTUNm82-Sardinia-1500-1700CE | 5 | 0.40 | 15 | 93 |
| aTUNm83-Sardinia-1500-1700CE | 11 | 0.05 | 2 | 89 |
| aTUNm84-Sardinia-1500-1700CE | 5 | 0.39 | 14 | 90 |
| aTUNm85-Sardinia-1500-1700CE | 13 | 0.56 | 34 | 81 |
| aTUNm86-Sardinia-1500-1700CE | 7 | 0.14 | 5 | 99 |
| aTUNm87-Sardinia-1500-1700CE | 14 | 0.58 | 58 | 93 |
| aTUNm88-Sardinia-1500-1700CE | 9 | 0.49 | 13 | 84 |
| aTUNm89-Sardinia-1500-1700CE | 6 | 0.57 | 15 | 80 |
| aTUNm90-Sardinia-1500-1700CE | 10 | 0.59 | 31 | 87 |
| aTUNm91-Sardinia-1500-1700CE | 3 | 0.50 | 11 | 90 |
| aTUNm92-Sardinia-1500-1700CE | 3 | 0.36 | 7 | 96 |

*Table S9: Summary statistics from Paleomix for the modern Atlantic bluefin specimens from Norway. The endogenous content is calculated from the alignment to the Atlantic bluefin nuclear reference genome.*

| **Sample-ID** | **Reads**  **(millions)** | **Endogenous DNA (fraction)** | **Mitochondrial**  **coverage** | **Mean fragment length (bp)** |
| --- | --- | --- | --- | --- |
| mTUNn01-NOR | 75 | 0.69 | 2784 | 178 |
| mTUNn02-NOR | 59 | 0.73 | 1019 | 170 |
| mTUNn03-NOR | 56 | 0.72 | 1508 | 172 |
| mTUNn04-NOR | 79 | 0.75 | 1150 | 178 |
| mTUNn05-NOR | 69 | 0.76 | 823 | 172 |
| mTUNn06-NOR | 79 | 0.75 | 1633 | 169 |
| mTUNn07-NOR | 73 | 0.77 | 1163 | 170 |
| mTUNn08-NOR | 76 | 0.75 | 1811 | 175 |
| mTUNn09-NOR | 90 | 0.76 | 1960 | 171 |
| mTUNn10-NOR | 93 | 0.75 | 2345 | 171 |
| mTUNn11-NOR | 21 | 0.76 | 273 | 168 |
| mTUNn12-NOR | 19 | 0.75 | 307 | 168 |
| mTUNn13-NOR | 23 | 0.78 | 296 | 168 |
| mTUNn14-NOR | 19 | 0.76 | 247 | 170 |
| mTUNn15-NOR | 18 | 0.76 | 283 | 166 |
| mTUNn16-NOR | 22 | 0.76 | 370 | 170 |
| mTUNn17-NOR | 22 | 0.75 | 260 | 168 |
| mTUNn18-NOR | 33 | 0.75 | 512 | 169 |
| mTUNn19-NOR | 22 | 0.77 | 228 | 167 |
| mTUNn20-NOR | 19 | 0.75 | 251 | 168 |
| mTUNn21-NOR | 19 | 0.76 | 520 | 168 |
| mTUNn22-NOR | 17 | 0.77 | 286 | 166 |
| mTUNn23-NOR | 17 | 0.77 | 263 | 167 |
| mTUNn24-NOR | 23 | 0.77 | 301 | 168 |
| mTUNn25-NOR | 23 | 0.75 | 381 | 169 |
| mTUNn26-NOR | 21 | 0.76 | 389 | 169 |
| mTUNn27-NOR | 25 | 0.76 | 343 | 168 |
| mTUNn28-NOR | 25 | 0.76 | 349 | 166 |
| mTUNn29-NOR | 22 | 0.75 | 448 | 168 |
| mTUNn30-NOR | 23 | 0.77 | 419 | 169 |
| mTUNn31-NOR | 27 | 0.77 | 687 | 166 |
| mTUNn32-NOR | 22 | 0.78 | 543 | 168 |
| mTUNn33-NOR | 22 | 0.75 | 458 | 169 |
| mTUNn34-NOR | 18 | 0.76 | 417 | 166 |
| mTUNn35-NOR | 24 | 0.76 | 472 | 167 |
| mTUNn36-NOR | 23 | 0.76 | 356 | 168 |
| mTUNn37-NOR | 24 | 0.77 | 419 | 166 |
| mTUNn38-NOR | 24 | 0.75 | 425 | 169 |

*Table S10: Summary statistics from Paleomix for the modern Atlantic bluefin specimens from the Mediterranean and the Gulf of Mexico. The endogenous content is calculated from the alignment to the Atlantic bluefin nuclear reference genome.*

| **Sample-ID** | **Reads**  **(millions)** | **Endogenous DNA (fraction)** | **Mitochondrial**  **coverage** | **Mean fragment length (bp)** |
| --- | --- | --- | --- | --- |
| mTUNm01-EMED | 24 | 0.68 | 716 | 112 |
| mTUNm02-EMED | 25 | 0.68 | 704 | 124 |
| mTUNm03-EMED | 22 | 0.69 | 336 | 118 |
| mTUNm04-EMED | 13 | 0.80 | 445 | 156 |
| mTUNm05-EMED | 48 | 0.48 | 2089 | 120 |
| mTUNm06-EMED | 37 | 0.49 | 1600 | 115 |
| mTUNm07-EMED | 31 | 0.67 | 708 | 105 |
| mTUNm08-EMED | 21 | 0.72 | 230 | 102 |
| mTUNm09-EMED | 19 | 0.71 | 363 | 100 |
| mTUNm10-EMED | 23 | 0.73 | 538 | 103 |
| mTUNm11-WMED | 24 | 0.68 | 1021 | 83 |
| mTUNm12-WMED | 27 | 0.70 | 660 | 112 |
| mTUNm13-WMED | 14 | 0.59 | 574 | 104 |
| mTUNm14-WMED | 20 | 0.70 | 396 | 81 |
| mTUNm15-WMED | 26 | 0.66 | 840 | 119 |
| mTUNm16-WMED | 46 | 0.61 | 1462 | 80 |
| mTUNm17-WMED | 63 | 0.58 | 1212 | 67 |
| mTUNm18-WMED | 60 | 0.57 | 1183 | 62 |
| mTUNm19-WMED | 41 | 0.69 | 410 | 95 |
| mTUNm20-WMED | 74 | 0.65 | 460 | 70 |
| mTUNm31-CMED | 36 | 0.35 | 1180 | 122 |
| mTUNm32-CMED | 32 | 0.30 | 666 | 126 |
| mTUNm33-CMED | 54 | 0.60 | 1385 | 69 |
| mTUNm34-CMED | 56 | 0.34 | 976 | 128 |
| mTUNm35-CMED | 38 | 0.61 | 448 | 71 |
| mTUNm36-CMED | 32 | 0.71 | 745 | 106 |
| mTUNm37-CMED | 45 | 0.55 | 530 | 104 |
| mTUNm38-CMED | 36 | 0.35 | 176 | 128 |
| mTUNm39-CMED | 62 | 0.58 | 705 | 81 |
| mTUNm40-CMED | 51 | 0.58 | 533 | 60 |
| mTUNm41-GOM | 80 | 0.66 | 696 | 69 |
| mTUNm42-GOM | 51 | 0.38 | 972 | 111 |
| mTUNm43-GOM | 30 | 0.49 | 565 | 106 |
| mTUNm44-GOM | 52 | 0.56 | 881 | 100 |
| mTUNm45-GOM | 44 | 0.47 | 966 | 128 |
| mTUNm46-GOM | 26 | 0.69 | 319 | 78 |
| mTUNm47-GOM | 46 | 0.69 | 49 | 75 |
| mTUNm48-GOM | 26 | 0.65 | 34 | 67 |
| mTUNm49-GOM | 67 | 0.55 | 910 | 102 |
| mTUNm50-GOM | 53 | 0.60 | 430 | 66 |

*Table S11: Summary statistics from Paleomix for the modern albacore specimens from the Bay of Biscay. The endogenous content is calculated from the alignment to the Atlantic bluefin nuclear reference genome.*

| **Sample-ID** | **Reads**  **(millions)** | **Endogenous DNA (fraction)** | **Mitochondrial**  **coverage** | **Mean fragment length (bp)** |
| --- | --- | --- | --- | --- |
| ALB-TUN-01 | 37 | 0.59 | 277 | 99 |
| ALB-TUN-02 | 19 | 0.66 | 219 | 117 |
| ALB-TUN-03 | 28 | 0.65 | 103 | 124 |
| ALB-TUN-04 | 27 | 0.66 | 377 | 74 |
| ALB-TUN-05 | 12 | 0.70 | 243 | 95 |
| ALB-TUN-06 | 25 | 0.68 | 109 | 99 |

*Table S12: Summary statistics from Paleomix for the modern Pacific bluefin specimens from the Nansei Islands. Whole genome raw sequence data downloaded from the DDBJ database (Kodama et al., 2012). The endogenous content is calculated from the alignment to the Atlantic bluefin nuclear reference genome.*

| **Sample-ID** | **Reads**  **(millions)** | **Endogenous DNA (fraction)** | **Mitochondrial**  **coverage** | **Mean fragment length (bp)** |
| --- | --- | --- | --- | --- |
| PBFT-TUN-01 | 121 | 0.72 | 2657 | 152 |
| PBFT-TUN-02 | 127 | 0.74 | 5107 | 151 |
| PBFT-TUN-03 | 109 | 0.76 | 2346 | 151 |
| PBFT-TUN-04 | 112 | 0.73 | 1361 | 151 |
| PBFT-TUN-05 | 107 | 0.76 | 4005 | 151 |
| PBFT-TUN-06 | 112 | 0.75 | 3345 | 151 |
| PBFT-TUN-07 | 99 | 0.76 | 3061 | 151 |
| PBFT-TUN-08 | 126 | 0.75 | 3465 | 151 |
| PBFT-TUN-09 | 114 | 0.74 | 4555 | 151 |

*Table S13: Samples with discordant MT haplotypes. Samples that were removed from further analyses due to being identical with other samples and/or having high missingness are marked with a star (*).*

| **Sample-ID** | **Mitochondrial haplotype** |
| --- | --- |
| aTUNn21-Norway-3000BCE | Pacific bluefin |
| aTUNn22-Norway-3000BCE | Pacific bluefin |
| aTUNn25-Norway-3000BCE * | Pacific bluefin |
| aTUNn26-Norway-3000BCE * | Pacific bluefin |
| aTUNn27-Norway-3000BCE * | Pacific bluefin |
| aTUNn28-Norway-3000BCE * | Pacific bluefin |
| aTUNn29-Norway-3000BCE * | Pacific bluefin |
| aTUNn33-Norway-3000BCE * | Pacific bluefin |
| aTUNn34-Norway-3000BCE * | Pacific bluefin |
| aTUNn36-Norway-3000BCE * | Pacific bluefin |
| aTUNm02-Sicily-900-1200CE | Albacore |
| aTUNm04-Sicily-900-1200CE * | Albacore |
| aTUNm06-Sicily-900-1200CE | Albacore |
| aTUNm39-Istanbul-800-1200CE | Pacific bluefin |
| aTUNm46-Istanbul-800-1200CE | Albacore |
| aTUNm81-Sardinia-1500-1700CE | Pacific bluefin |
| mTUNn05-NOR | Albacore |
| mTUNn17-NOR | Pacific bluefin |
| mTUNn35-NOR | Pacific bluefin |
| mTUNm15-WMED | Albacore |

*Table S14: Jointly called and filtered datasets, used in population genomic analyses (N = number of samples in each dataset).*

|  |  | Location | Subset Code | N |
| --- | --- | --- | --- | --- |
| Containing samples with discordant haplotypes | Modern | Norwegian sea | **NORAll** | 38 |
|  |  | Western Mediterranean | **WMEDAll** | 10 |
|  |  | All modern Atlantic bluefin locations | **modernABFT** | 78 |
|  | Ancient | Jortveit, Norway | **Norway_3000BCE_All** | 25 |
|  |  | Sardinia, Italy | **Sardinia_1500_1700CE_All** | 15 |
|  |  | Istanbul, Turkey | **Istanbul_800_1200CE_All** | 19 |
|  |  | Sicily, Italy | **Sicily_900_1200CE_All** | 10 |
|  |  | All ancient  excavation locations | **AncientAll** | 109 |
|  | Both modern and ancient | All modern and ancient Atlantic bluefin locations | **AllABFT** | 187 |
| No discordant haplotypes present | Modern | Norwegian sea | **NORExIntrog** | 35 |
|  |  | Eastern Mediterranean | **EMED** | 10 |
|  |  | Central Mediterranean | **CMED** | 10 |
|  |  | Western Mediterranean | **WMEDExIntrog** | 9 |
|  |  | Gulf of Mexico | **GOM** | 10 |
|  |  | All modern Atlantic bluefin locations | **modernExIntrog** | 74 |
|  | Ancient | Jortveit, Norway | **Norway_3000BCE_ExIntrog** | 23 |
|  |  | Sardinia, Italy | **Sardinia_1500_1700CE_ExIntrog** | 14 |
|  |  | Istanbul, Turkey | **Istanbul_800_1200CE_ExIntrog** | 17 |
|  |  | Sicily, Italy | **Sicily_900_1200CE_ExIntrog** | 8 |
|  |  | Cádiz, Spain | **Gibraltar_100CE** | 4 |
|  |  | Conil de la Frontera, Spain | **Gibraltar_1755CE** | 7 |
|  |  | Istanbul, Turkey | **Istanbul_1941CE** | 1 |
|  |  | Marseille, France | **Marseille_1800CE** | 8 |
|  |  | Zliten, Libya | **Zliten_1925CE** | 20 |
|  |  | All ancient  excavation locations | **AncientExIntrog** | 102 |
|  | Both modern and ancient | All modern and ancient Atlantic bluefin locations | **AllExIntrog** | 176 |
| Datasets with jointly called and filtered outgroup species | All samples in **AllABFT**  + All albacore samples  + All Pacific bluefin samples | All Atlantic bluefin locations  + Bay of Biscay (albacore)  + Nansei Islands (Pacific bluefin) | **All_ALB_PBFT** | 202 |

In the table above, the following datasets were used for population genomic analyses:

- AllABFT: Used for LociMissingness analyses (Figure S5) and calculation of ΦST and d_xy_ (Figure 3B). No outgroup.
- All_ALB_PBFT: Used for interspecific PCA (Figure 2A) with no outgroup. It was also used for haplotype network (Figure 2C) and ML and Bayesian phylogenies (Figure 2D and Figure S6) using one katsuwonus pelamis as outgroup (NCBI Reference Sequence: NC_005316.1)
- AllExIntrog: Used for intraspecific PCA (Figure S8) and calculation of ΦST and d_xy_ (Figure 3A) with no outgroup. It was also used for intraspecific haplotype network (Figure S9), as well as ML and Bayesian phylogenies (Figure S10) using one Pacific bluefin as outgroup (NCBI Reference Sequence: NC_008455.1)

All other datasets were only used for the calculation of population genomic statistics (Table S15).

*Table S15: Population genomic statistics of the separately called and filtered datasets. N = number of samples, hD = haplotype diversity, Nh = number of haplotypes, S = number of segregating sites, π = nucleotide diversity, TD = Tajima’s D. Significance levels (**=0.01, *=0.05, n.s.=not significant) are indicated for the TD values. Datasets containing discordant haplotypes have been highlighted in beige color.*

| **Subset** | **N** | **hD** | **Nh** | **S** | **π** | **TD** | **π(bs)** | **TD(bs)** |
| --- | --- | --- | --- | --- | --- | --- | --- | --- |
| AllABFT_mt | 186 | 0.998 | 160 | 809 | 0.0033 | -3.23 (**) | 0.0027 | -2.49 |
| AllExIntrog_mt | 175 | 0.999 | 158 | 475 | 0.0009 | -3.23 (**) | 0.0007 | -2.82 |
| modernABFT_mt | 78 | 0.998 | 72 | 699 | 0.0035 | -2.14 (*) | 0.0035 | -1.18 |
| modernExIntrog_mt | 74 | 0.999 | 70 | 300 | 0.0012 | -2.49 (*) | 0.0011 | -1.20 |
| AncientAll_mt | 108 | 0.998 | 97 | 710 | 0.0034 | -3.37 (**) | 0.0024 | -3.42 |
| AncientExIntrog_mt | 101 | 0.997 | 90 | 332 | 0.0008 | -3.37 (**) | 0.0005 | -3.45 |
| NORAll_mt | 38 | 0.997 | 36 | 621 | 0.0049 | -1.73 (n.s.) | 0.0050 | -0.92 |
| NORExIntrog_mt | 35 | 0.998 | 34 | 187 | 0.0012 | -2.10 (*) | 0.0012 | -0.85 |
| WMEDAll_mt | 10 | 1.000 | 10 | 477 | 0.0060 | -2.14 (*) | 0.0060 | -1.28 |
| WMEDExIntrog_mt | 9 | 1.000 | 9 | 74 | 0.0011 | -1.71 (n.s.) | 0.0011 | -1.21 |
| EMED_mt | 10 | 1.000 | 10 | 90 | 0.0013 | -1.59 (n.s.) | 0.0013 | -0.93 |
| CMED_mt | 10 | 1.000 | 10 | 90 | 0.0012 | -1.87 (n.s.) | 0.0012 | -1.21 |
| GOM_mt | 10 | 1.000 | 10 | 78 | 0.0010 | -2.17 (*) | 0.0010 | -1.58 |
| Norway_3000BCE_All | 24 | 1.000 | 24 | 556 | 0.0046 | -3.91 (**) | 0.0029 | -3.68 |
| Norway_3000BCE_ExIntrog | 23 | 1.000 | 22 | 201 | 0.0012 | -3.92 (**) | 0.0008 | -3.65 |
| Sardinia_1500_1700CE_All | 15 | 1.000 | 15 | 504 | 0.0042 | -3.31 (**) | 0.0035 | -2.46 |
| Sardinia_1500_1700CE_ExIntrog | 14 | 1.000 | 14 | 95 | 0.0009 | -3.47 (**) | 0.0007 | -2.65 |
| Istanbul_800_1200CE_All | 19 | 1.000 | 19 | 542 | 0.0054 | -2.90 (**) | 0.0049 | -1.94 |
| Istanbul_800_1200CE_ExIntrog | 17 | 1.000 | 17 | 85 | 0.0009 | -3.04 (**) | 0.0007 | -2.01 |
| Sicily_900_1200CE_All | 10 | 1.000 | 10 | 411 | 0.0062 | -4.18 (**) | 0.0037 | -4.15 |
| Sicily_900_1200CE_ExIntrog | 8 | 1.000 | 8 | 43 | 0.0006 | -4.55 (**) | 0.0003 | -4.64 |
| Gibraltar_100CE | 4 | 1.000 | 4 | 27 | 0.0008 | -0.63 (n.s.) | n.a. | n.a. |
| Gibraltar_1755CE | 7 | 1.000 | 7 | 30 | 0.0003 | -5.17(**) | 0.0001 | -6.27 |
| Istanbul_1941CE | 1 | n.a. | 1 | 0 | n.a. | n.a. | n.a. | n.a. |
| Marseille_1800CE | 8 | 1.000 | 8 | 40 | 0.0006 | -2.96 (**) | 0.0005 | -2.70 |
| Zliten_1925CE | 20 | 1.000 | 20 | 111 | 0.0008 | -3.75 (**) | 0.0006 | -3.38 |

Section 3: Supplementary Figures


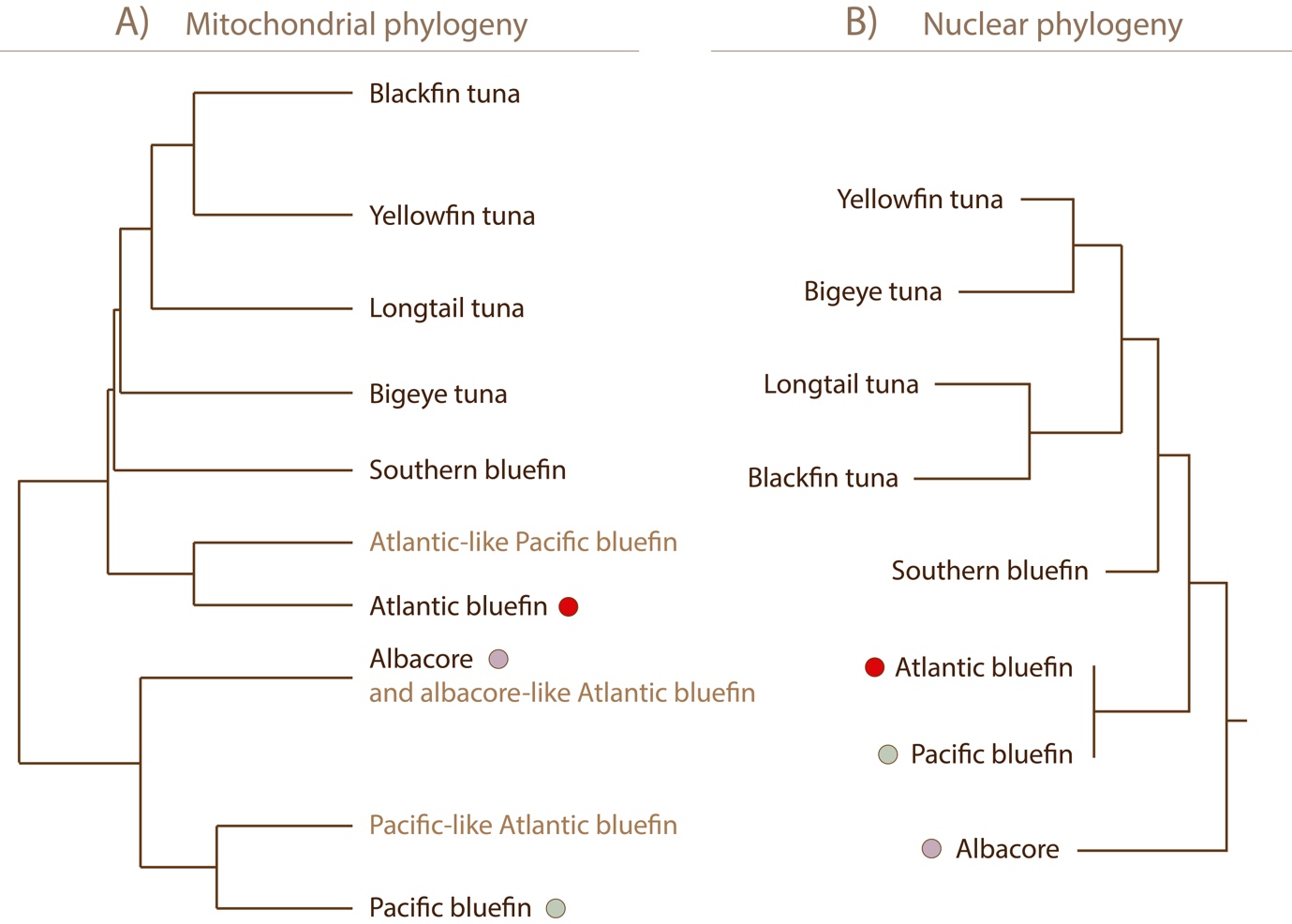


*Figure S1: Thunnus phylogenies based on A) the mitochondrial control region adapted from Viñas & Tudela (2009) and B) genome-wide nuclear markers adapted from Díaz-Arce et al. (2016). Tip labels in light brown represent discordant MT haplotypes. The colored circles mark species presented in this study.*


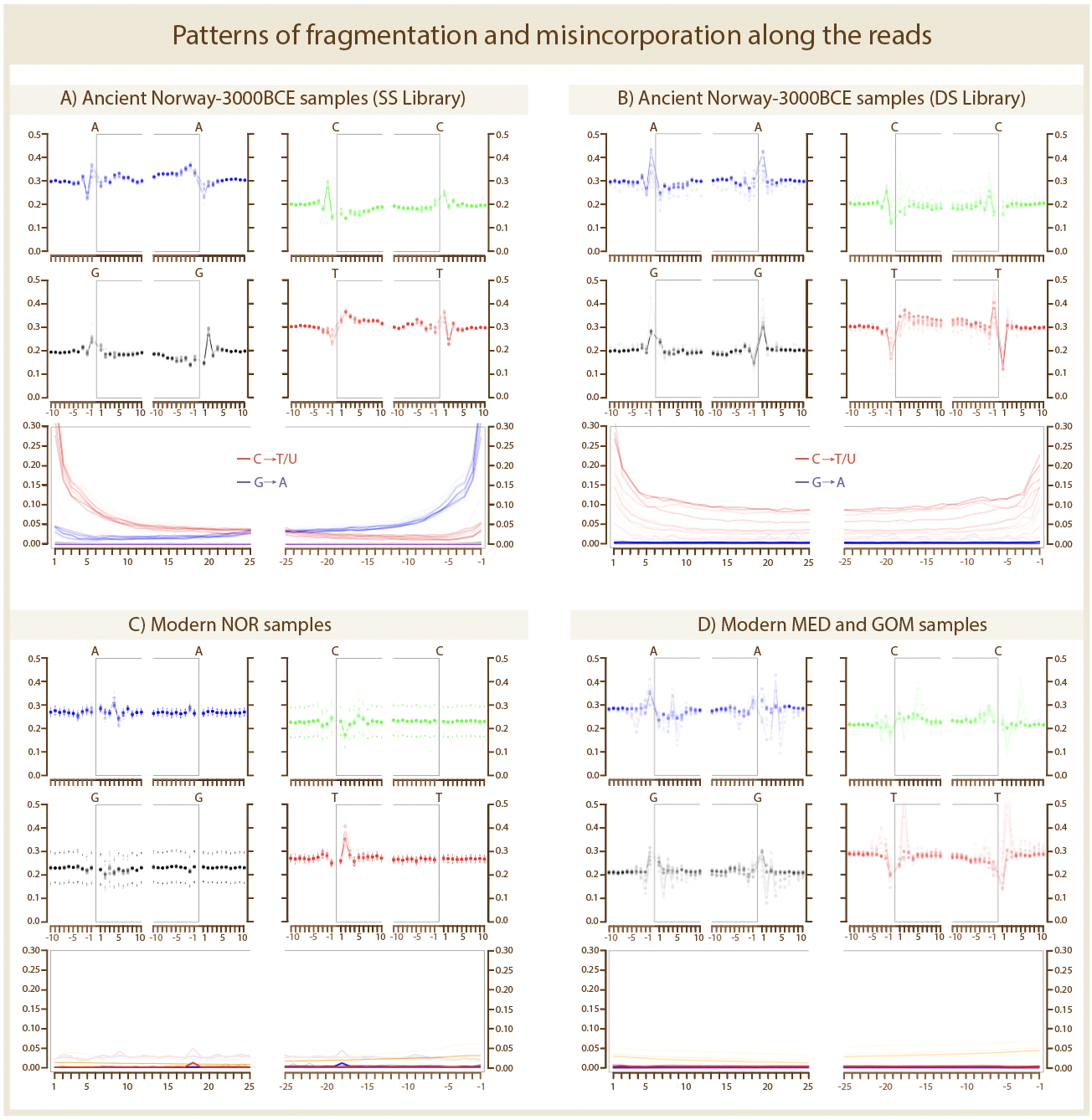


*Figure S2: Fragmentation (upper panels) and misincorporation (lower panels) plots from mapDamage v.2.0.9. The fragmentation plots show the base frequency inside and surrounding the read, where the grey box indicates the location of where the reads have mapped to the reference. The misincorporation plots show the rate of substitutions along the positions of the read ends, relative to the reference (Red: C to T. Blue: G to A. Grey: All other substitutions. Green: Deletions. Purple: Insertions. Orange: Soft-clipped bases).*


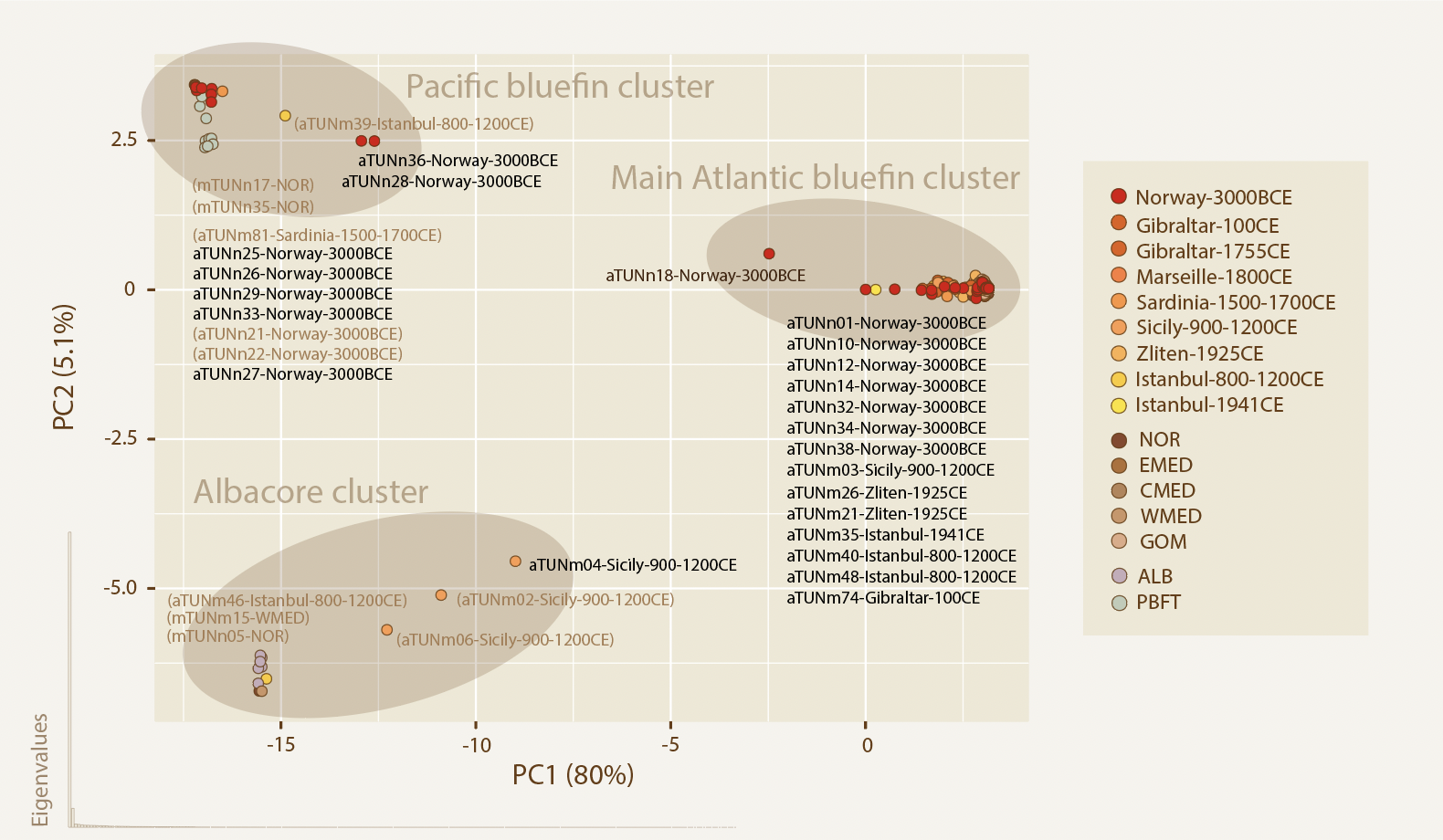


*Figure S3: PCA of all samples included in the exploratory analysis, prior to omission of identical- and high missingness samples. The plot shows an interspecific PCA. Samples that were excluded from subsequent population genomic analyses are marked with the sample name in dark brown. Sample names in parentheses indicate diverging samples that were kept, but taken into account as discordant haplotypes, in the population genomic analyses.*


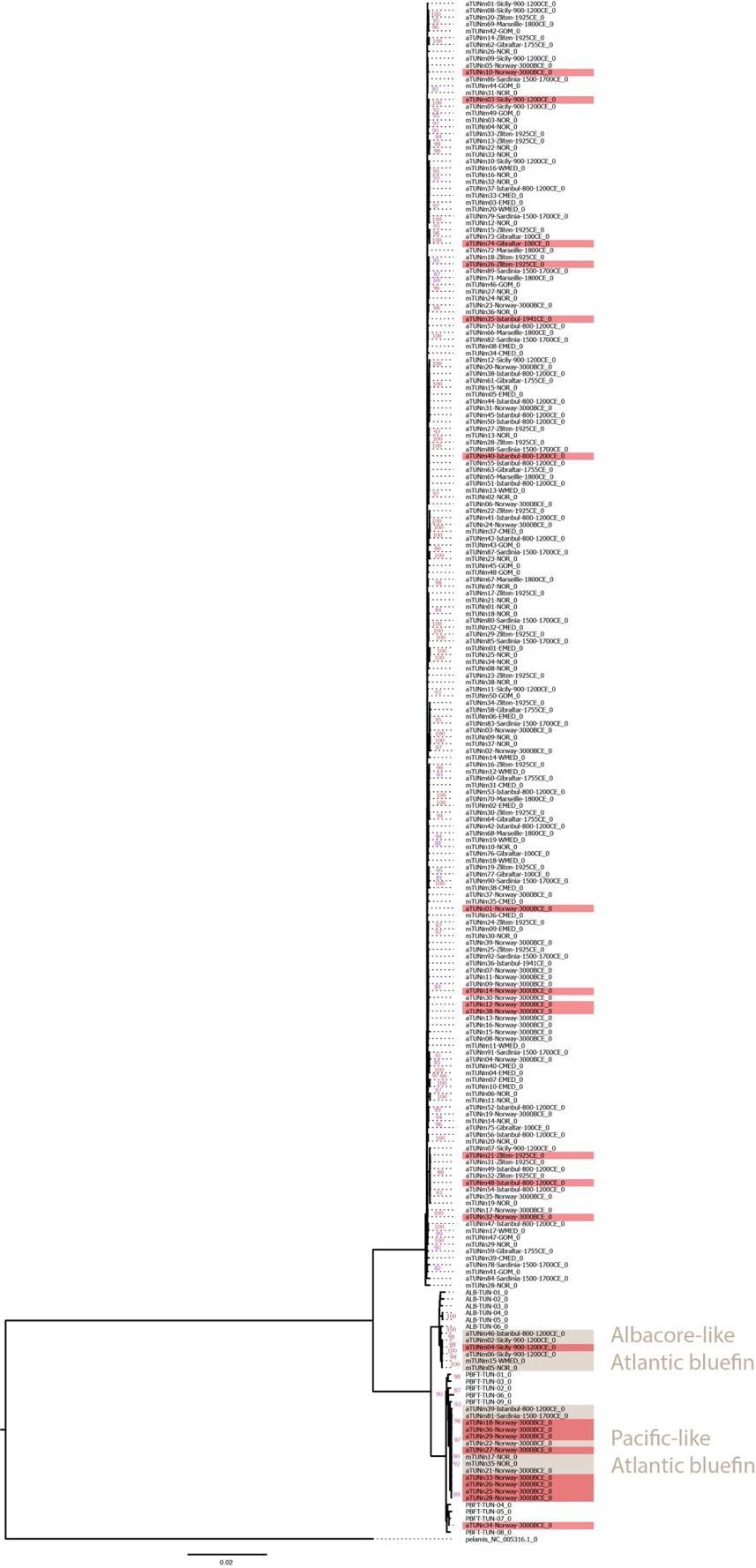


*Figure S4: ML phylogeny of all samples included in the exploratory analysis, prior to omission of identical- and high missingness samples. Bootstrap values over 80 are shown in pink. Samples that were excluded from subsequent population genomic analyses are highlighted in red. Samples highlighted in brown indicate diverging samples that were kept, but taken into account as discordant haplotypes, in the population genomic analyses.*


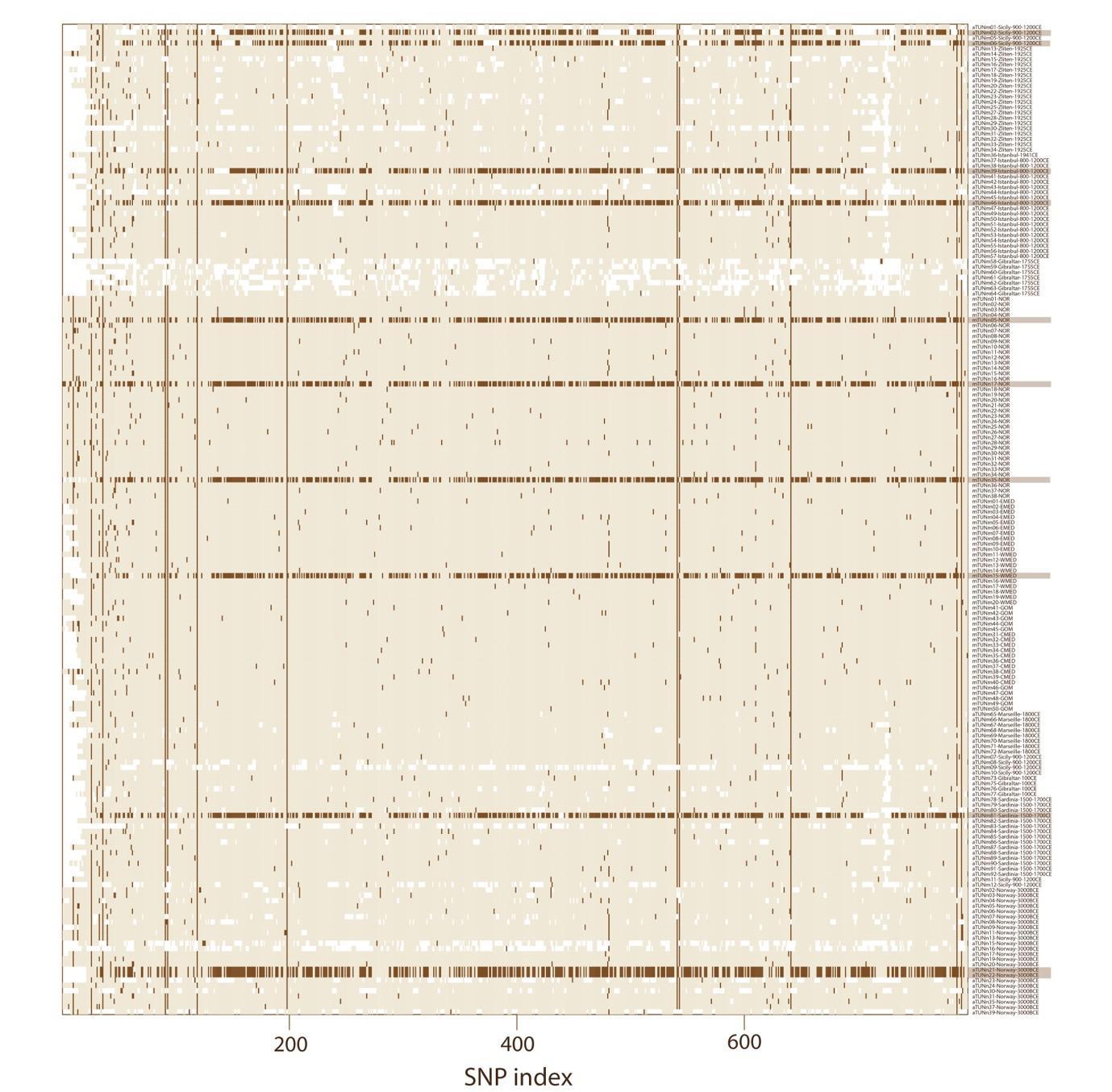


*Figure S5: Missing loci (white) and the presence of second variants (dark brown) across the Atlantic bluefin MT genomes (dataset: AllABFT). The 11 specimens that were identified with discordant MT genomes have a high number of divergent variants when compared to the Atlantic bluefin reference genome.*


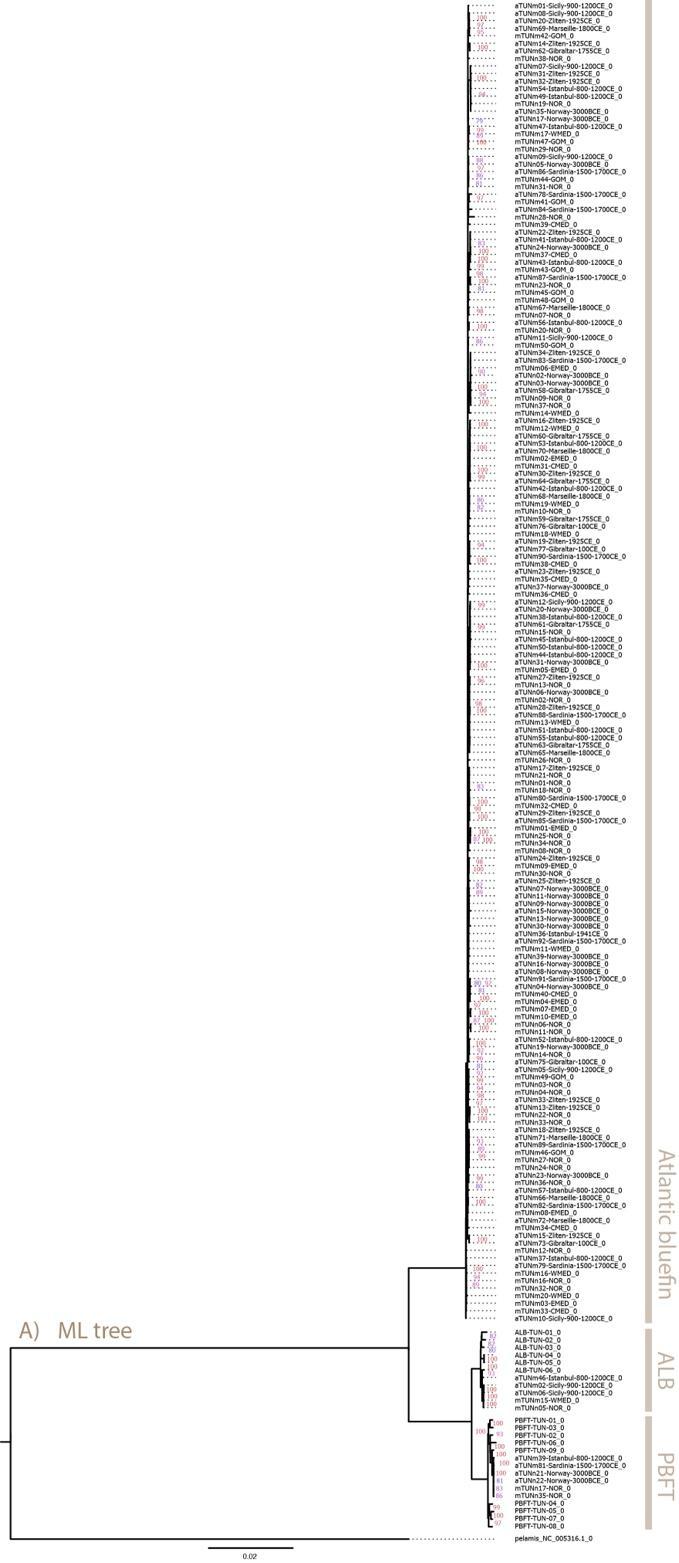

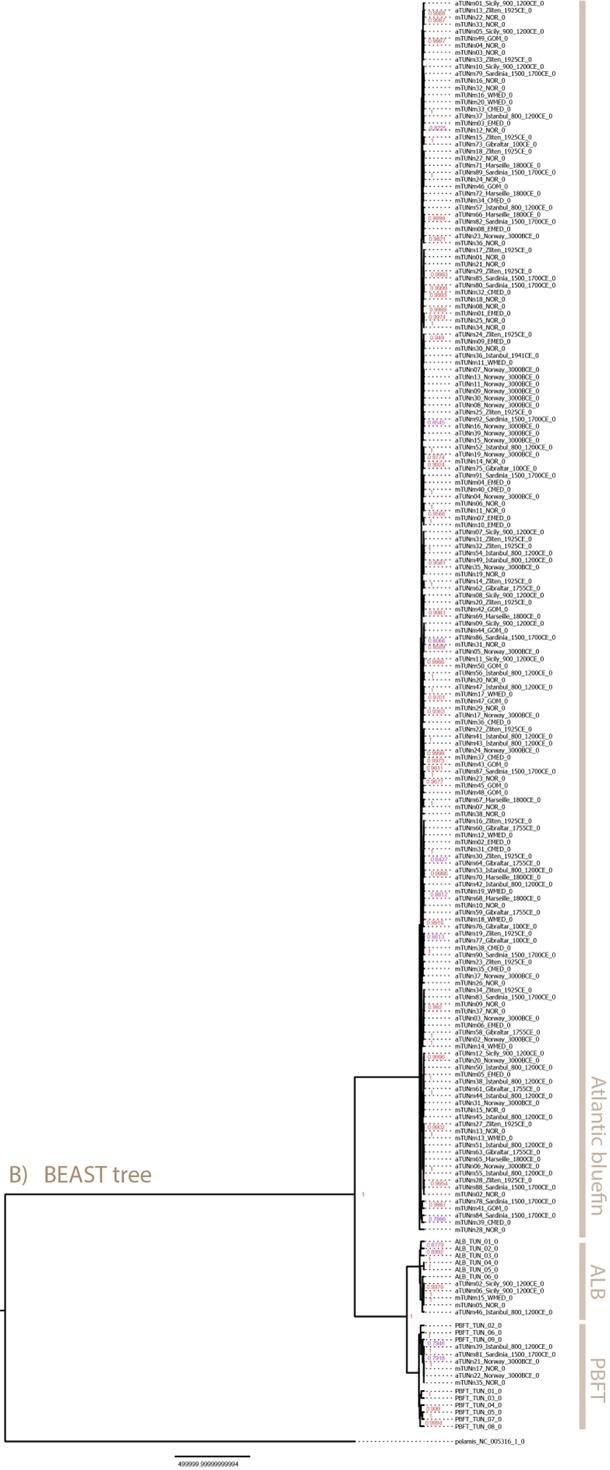


*Figure S6: ML and Bayesian phylogenies of all samples included in population genomic analyses (dataset: All_ALB_PBFT) using Skipjack tuna (Katsuwonus pelamis) as outgroup. Bootstrap values over 80 and posterior probability values over 0.8 are shown in pink in A) and B) respectively. Species clades are highlighted in brown.*


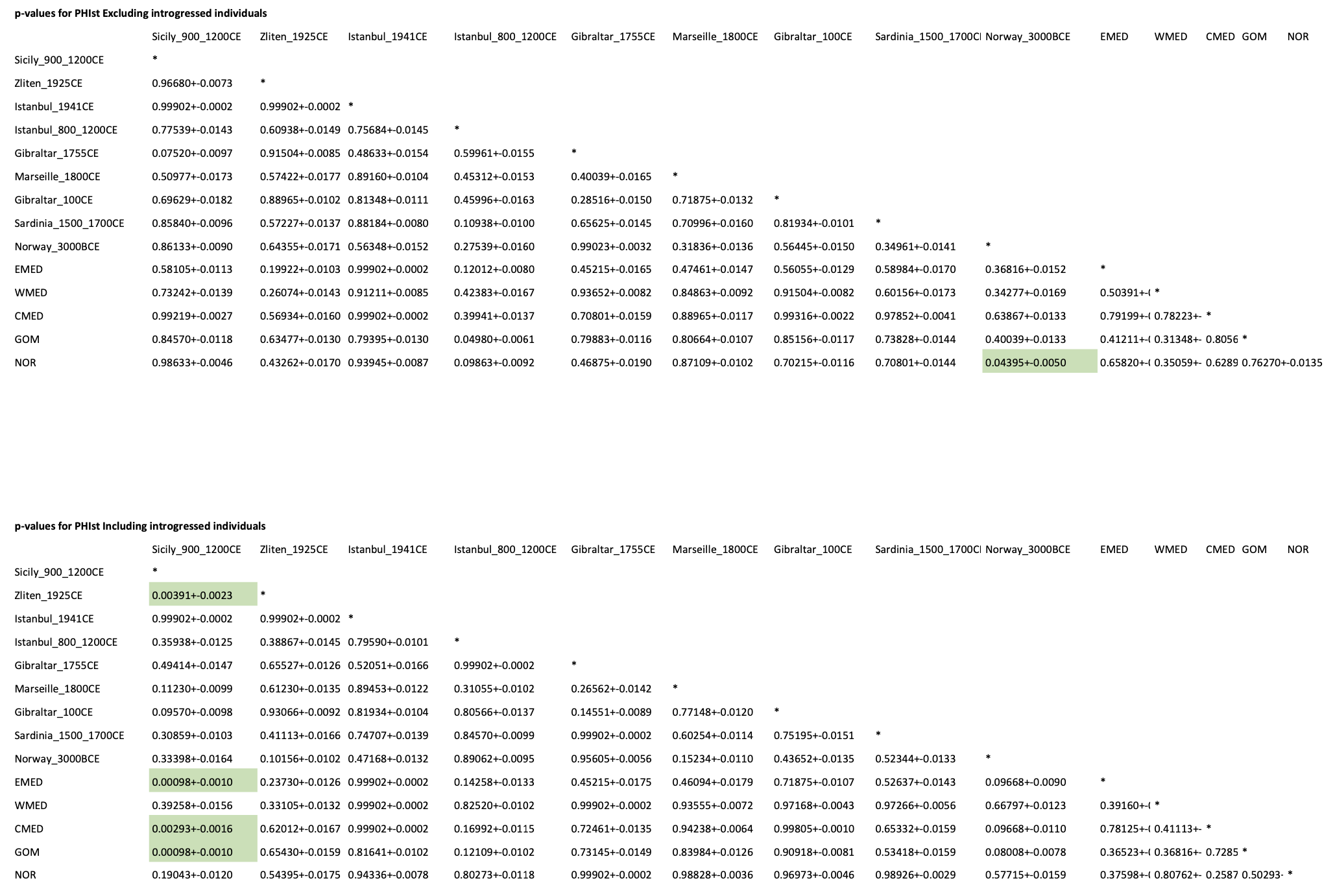


*Figure S7: ΦST P-values from Arlequin for the AllExIntrog (excluding discordant haplotypes) and AllABFT (including discordant haplotypes) datasets, corresponding to Figure 3A) and 3B) respectively.* *Significant p-values are marked in green. After correcting for multiple testing (Bonniferoni correction), none of the p-values remained significant.*


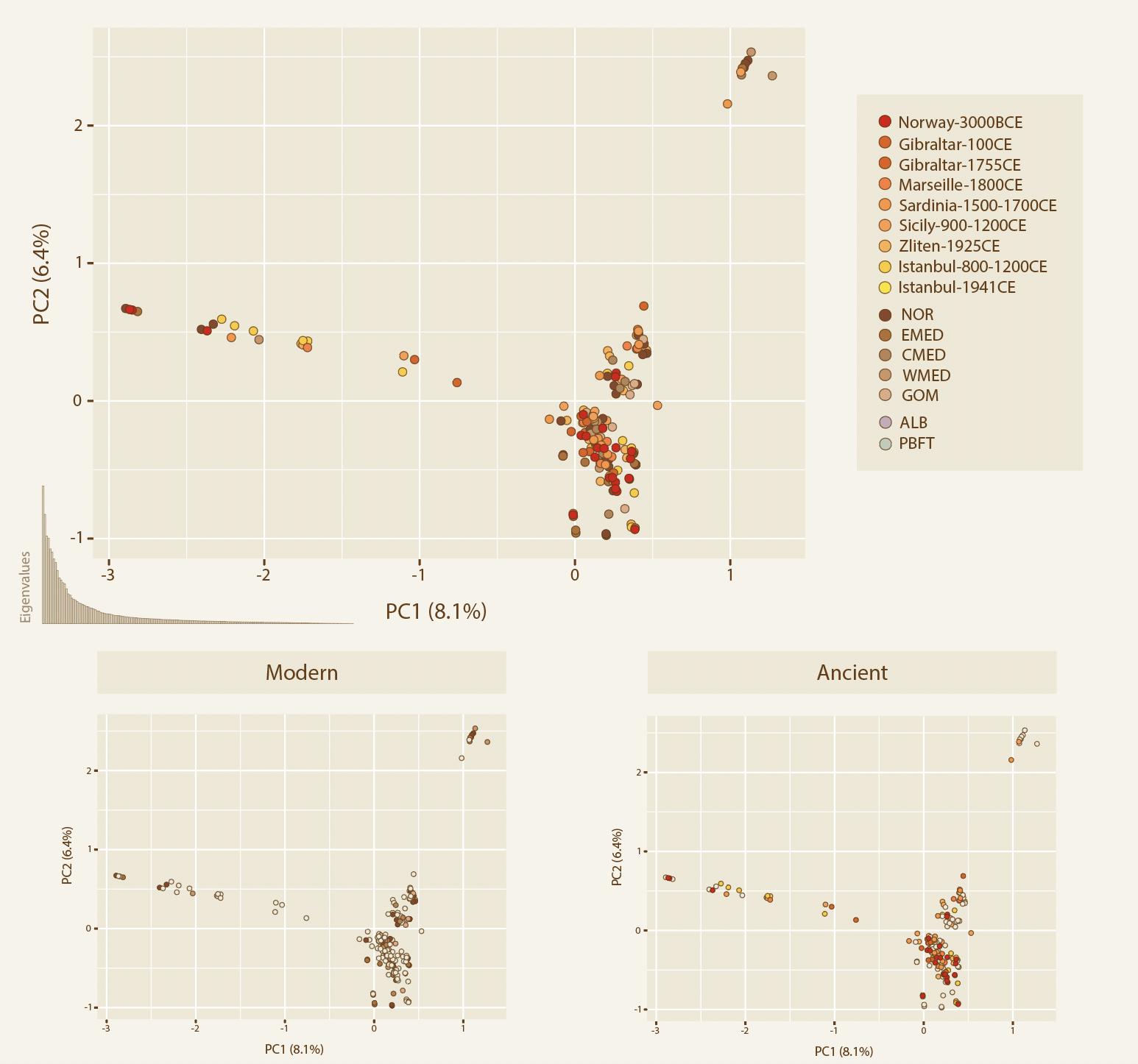


*Figure S8: PCA of all Atlantic bluefin-like haplotypes (dataset: AllExIntrog). The ancient and modern samples are highlighted in the bottom panel. Eigenvalues are shown in the left corner of the upper panel.*


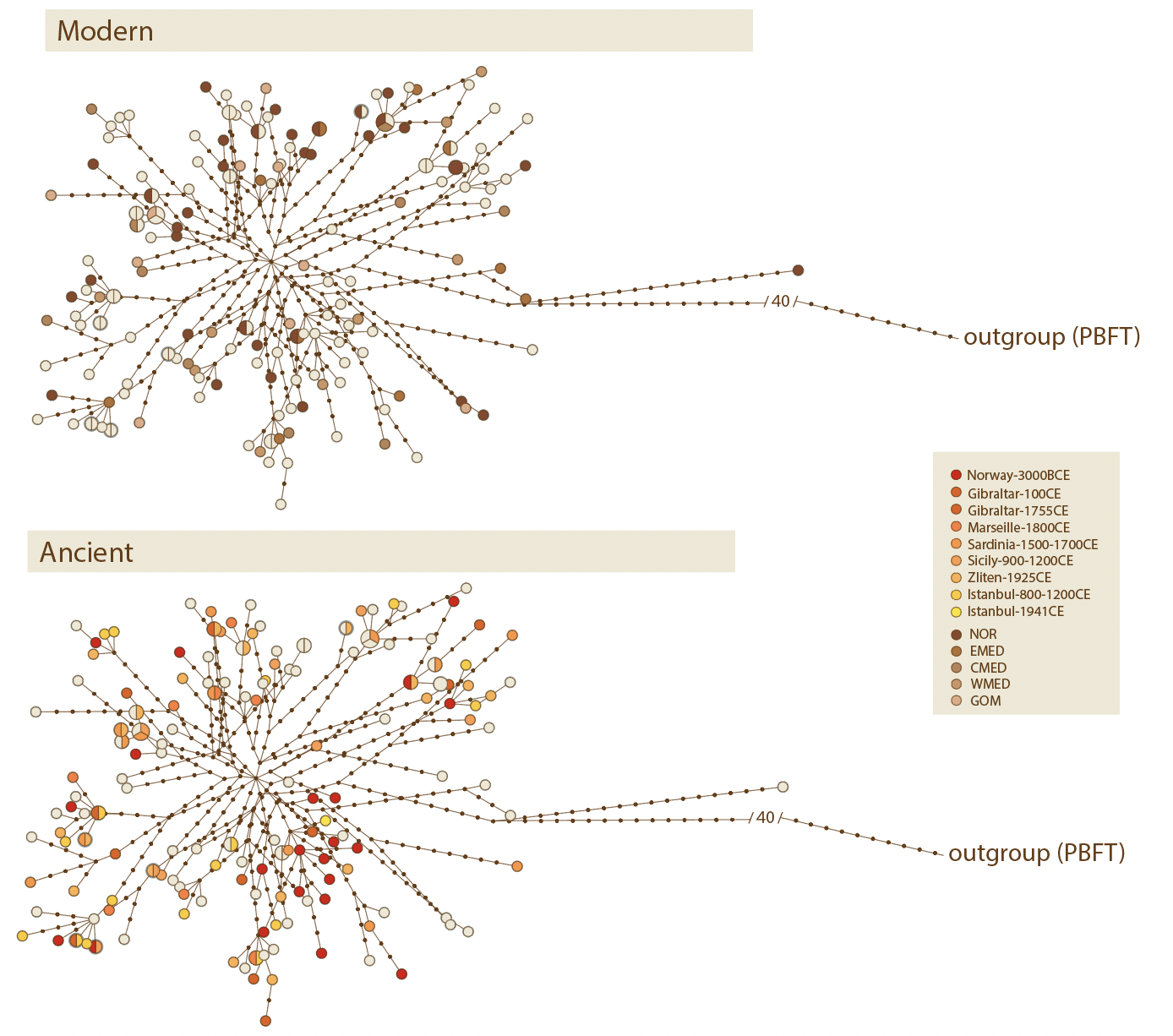


*Figure S9: Haplotype network of all Atlantic bluefin-like haplotypes, using dataset AllExIntrog (excluding discordant haplotypes) and Pacific bluefin (Thunnus orientalis) as outgroup. Each node represents a unique haplotype.*


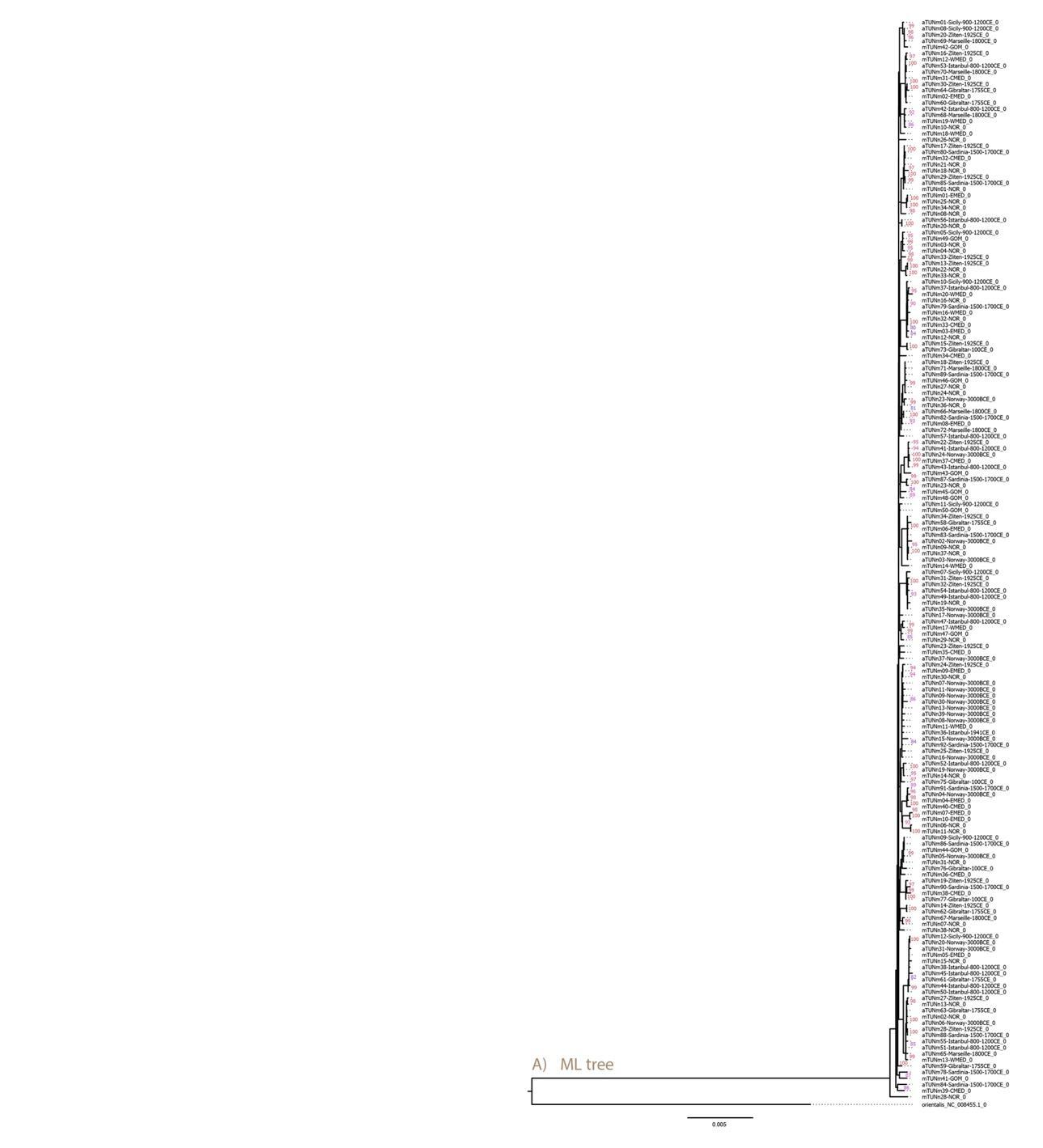

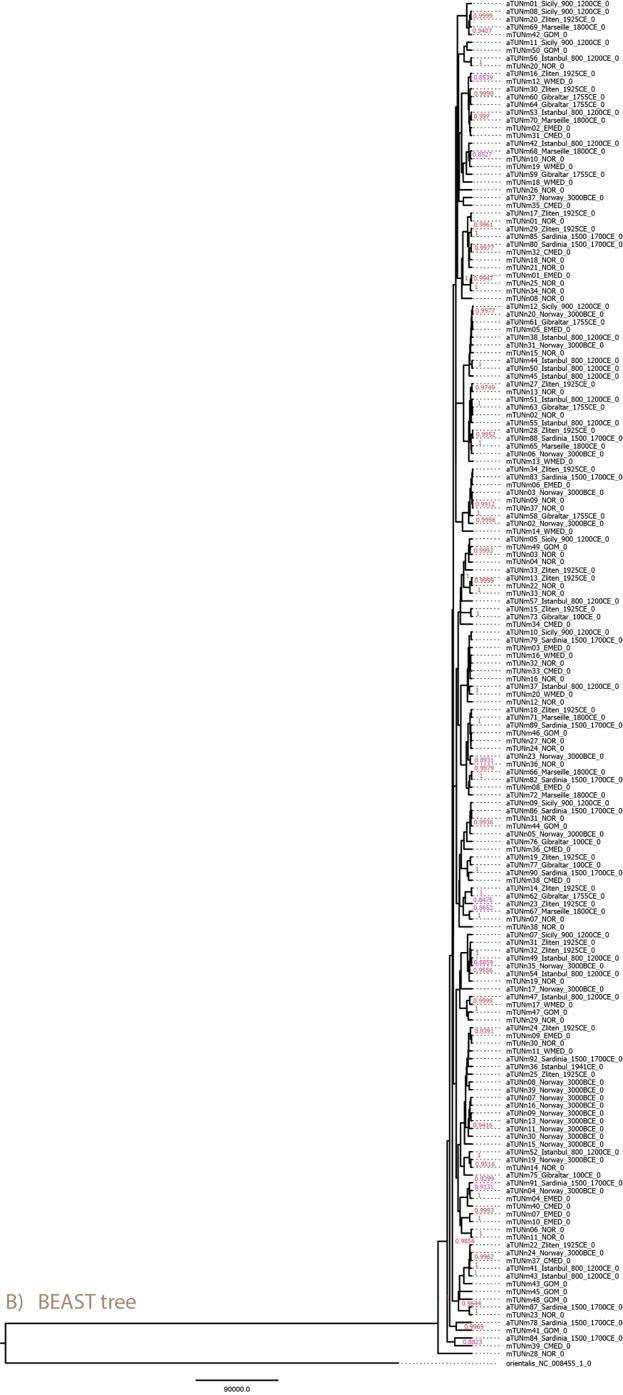


*Figure S10: ML and Bayesian phylogenies of all samples included in population genomic analyses (dataset: AllExIntrog) using Pacific bluefin (Thunnus orientalis) as outgroup. Bootstrap values over 80 and posterior probability values over 0.8 are shown in pink in A) and B) respectively.*
